# Etiology of acute meningitis and encephalitis from hospital-based surveillance in South Kazakhstan oblast, February 2017—January 2018

**DOI:** 10.1371/journal.pone.0251494

**Published:** 2021-05-14

**Authors:** Yekaterina Bumburidi, Gulmira Utepbergenova, Bakhtygali Yerezhepov, Nursulu Berdiyarova, Kaldikul Kulzhanova, Jennifer Head, Daphne Moffett, Daniel Singer, Pawan Angra, Toni Whistler, James Sejvar

**Affiliations:** 1 Central Asian Regional Office, Centers for Disease Control and Prevention, Almaty, Kazakhstan; 2 Shymkent City Infectious Disease Hospital–Regional Department of Health Care, Southern Kazakhstan Region, Kazakhstan, Shymkent; 3 Department of Infectious Diseases and Phthisiatry, Khoja Akhmet Yassawi International Kazakh-Turkish University, Kazakhstan, Turkestan; 4 Division of Global Health Protection, Centers for Disease Control and Prevention, Atlanta, GA, United States of America; 5 Public Health Institute, Oakland, CA, United States of America; 6 Association of Schools and Programs of Public Health, Washington DC, United States of America; 7 Division of High-Consequence Pathogens and Pathology, Centers for Disease Control and Prevention, Atlanta, GA, United States of America; Institut Pasteur, FRANCE

## Abstract

Encephalitis and meningitis (EM) are severe infections of the central nervous system associated with high morbidity and mortality. The etiology of EM in Kazakhstan is not clearly defined, so from February 1, 2017 to January 31, 2018 we conducted hospital-based syndromic surveillance for EM at the Shymkent City Hospital, in the South Kazakhstan region. All consenting inpatients meeting a standard case definition were enrolled. Blood and cerebrospinal fluid (CSF) samples were collected for bacterial culture, and CSF samples were additionally tested by PCR for four bacterial species and three viruses using a cascading algorithm. We enrolled 556 patients. Of these, 494 were of viral etiology (including 4 probable rabies cases), 37 were of bacterial etiology, 19 were of unknown etiology and 6 were not tested. The most commonly identified pathogens included enterovirus (73%, n = 406 cases), herpes simplex virus (12.8%, n = 71), and *Neisseria meningitidis* (3.8%, n = 21). The incidence rates (IRs) for enteroviral and meningococcal EM were found to be 14.5 and 0.7 per 100,000 persons, respectively. The IR for bacterial EM using both PCR and culture results was 3–5 times higher compared to culture-only results. Antibacterial medicines were used to treat 97.2% (480/494) of virus-associated EM. Incorporation of PCR into routine laboratory diagnostics of EM improves diagnosis, pathogen identification, ensures IRs are not underestimated, and can help avoid unnecessary antibacterial treatment.

## Introduction

Encephalitis and meningitis (EM) are infections of the central nervous system (CNS) often associated with substantial morbidity and mortality [1,2]. Meningitis, or inflammation of the meninges, is often self-limited and benign, but is up to 10 times more common than encephalitis, which is defined by inflammation of the brain parenchyma in conjunction with clinical signs of neurological dysfunction. A wide range of infectious agents, including viruses, bacteria, fungi, and parasites [3,4] cause infections of the CNS, often entering the CNS from the periphery through multiple mechanisms [2]. In addition, EM may arise from noninfectious causes (i.e. autoimmune, neoplastic-related, drug-induced, post-procedural, or systemic illnesses) [4]. Determining the etiology of EM is essential for understanding regional variation, for guiding treatment, and for prioritizing preventative strategies in public health [3,4].

In 2016 neurological disorders led to 276 million disability-adjusted life years (DALYs) and 9.0 million deaths, constituting the first and second ranked causes from the global disease burden, respectively. Meningitis contributed 7.9% to neurological DALYs after stroke, migraines, Alzheimer and other dementias [5]. The incidence rates (IRs) and causative agents of EM differ by geographic region. In the UK, the annual IR of viral and bacterial meningitis in adults are 2.73 and 1.24 per 100,000 persons respectively, with enteroviruses (EV) and herpes simplex viruses (HSV) being the predominant viral agents and *Streptococcus pneumoniae* the main bacterium [6]. In Ghana, located in West Africa’s “meningitis belt”, the incidence of meningitis ranges by district from 6.1 to 47.5 per 100,000, mainly caused by *S*. *pneumoniae* and *Neisseria meningitidis*, with a case fatality rate (CFR) of 12.2% [7]. Globally, EV are the leading cause of aseptic viral EM are commonly associated with meningitis outbreaks [8–13].

Meta-analysis of 25 studies shows that the annual incidence rate of suspected infectious encephalitis globally ranges from 1.5 to 7 cases per 100,000 population and the etiology of encephalitis is confirmed in 27.5% -79% cases. Excluding encephalitis causative agents, the occurrence of which are limited the natural endemic areas of the diseases, HSV and VZV are the most common agents of encephalitis worldwide [14]. Sporadic acute encephalitis caused by HSV presented worldwide with low estimated incidence of 0.22, 0.46 and 0.64 cases per 100,000 adults in Sweden, USA, and Denmark, respectively [15–17]. The CFR for encephalitis caused by HSV ranges from 5–15% in Sweden, France, and Turkey [17–19]. Both encephalitis and meningitis can result in death or other long-term disabilities in both children and adults, including permanent brain and nerve damage manifesting as hearing and/or speech loss, blindness, behavioral changes, cognitive disabilities, lack of muscle control, seizures, and cognitive impairment [3,20–23]. The ongoing morbidity in children further results in disproportionate public health and societal impact.

Currently, Kazakhstan’s surveillance system is designed to characterize EM of bacterial etiology only, with the existing diagnostic algorithm limited to culture of cerebral spinal fluid for *N*. *meningitidis* and other bacteria. Surveillance for viral EM is minimal, with testing limited to tick-borne encephalitis virus (TBEV) within naturally occurring endemic areas.

The goal of this project was to describe the epidemiology and potential etiology of EM cases and to show the value of expanded diagnostic testing of EM cases in South Kazakhstan Oblast (SKO), a region suspected of having among the highest burden of EM in the country. For this investigation, we incorporated laboratory assays for EVs, HSVs, VZV and bacterial pathogens which are commonly presented globally into the existing diagnostic testing algorithm for EM surveillance. We collected data from February 1, 2017 to January 31, 2018.

## Methods

### Ethical considerations

This study was approved by the Institution Review Board in Almaty, Kazakhstan (Protocol №: IRB-A072, November 2016) through the Committee for Public Health Protection of the Ministry of Health (Approval letter №35-2/689, February 2017). The protocol was reviewed in accordance with the United States Centers for Disease Control and Prevention human subjects review procedures (CGH 2017–004). Informed consent or assent was obtained verbally by attending physician from each participant aged 18 years and older, and from the parent or guardian if the patient was under 18 years old, or if s/he was deceased or unresponsive. A physician signed consent form was included in the patients’ medical record. No personal identifying information was collected.

### Study site

In this study, we selected SKO which is one of 14 oblasts (geopolitical regions) in Kazakhstan and located on the border of Kazakhstan and Uzbekistan, due to the high number of suspected EM cases. According to the Department of Public Health surveillance data, in 2017 SKO had a population of 2,898,090 including 1,008,894 children <15 years and 72,188 < 1 year old of which one-third resided in the regional capital, Shymkent. Population data were available by ages for children <15 years (one-year age groups), for adults ≥15 (five-year age groups); and also by organizational status for children 0–6 years old (enrollment/non-enrollment in pre-school/day-care institutions (PS/DCI)), and for 6 to17 years old enrolled in schools. There were 159,484 children enrolled in PS/DCI, 335,364 children were non-enrolled and 604,984 were enrolled in schools. In this study, children are defined of age <15 years old and adults ≥15years. We analyzed data by age groups and additionally by organizational status for EV cases.

Following existing Kazakhstan Clinical Protocols, all suspected EM cases are required to be hospitalized. We conducted prospective EM surveillance at the Shymkent City Infectious Disease Hospital, the main regional referral hospital with a 390-bed capacity. Enrollment covered a full 12-month period from February 1, 2017 to January 31, 2018. All participating medical doctors were trained in the case definition of EM and case management. Case data was verified on a weekly basis by the Deputy Chief of the referral hospital.

### Case definitions

For the encephalitis case definition, patients must have exhibited encephalopathy (defined as depressed or altered level of consciousness, lethargy or personality change lasting over 24 hours), and at least two of the following criteria: 1) documented fever ≥38°C within 72 h before or after presentation; 2) generalized or partial seizure not attributable to a preexisting seizure disorder; 3) new onset of focal neurological findings, such as focal cortical signs, cranial nerve abnormalities, visual field defects, presence of primitive reflexes (Babinski sign, glabellar reflex, snout/sucking reflex), motor weakness, sensory abnormalities, altered deep tendon reflexes, or cerebellar dysfunction (ataxia, dysmetria or cerebellar nystagmus); or 4) a cerebral spinal fluid (CSF) white blood cell (WBC) count >5 WBC x10^6^/L in children over 2 months of age and >15 WBC x10^6^/L in children under 2 months of age [24,25].

The standard case definition for meningitis included pleocytosis in the CSF determined as >5 leukocytes x10^6^/L in patients ≥2 months of age or >15 leukocytes x10^6^/L in patients <2 months of age, and any one of the following: 1) documented fever ≥38°C within 72 h before or after presentation; 2) headache; 3) vomiting; 4) bulging fontanelle; 5) other signs of meningeal irritation such as nuchal rigidity, Kernig’s sign, or Brudzinski’s sign; or 6) be hospitalized with the diagnosis of acute meningitis or meningococcemia [26]. Patients who met case definitions for both meningitis and encephalitis were identified as meningoencephalitis.

### Participant selection and recruitment

Patients admitted to the hospital during the study period with suspected EM were evaluated using the study case definition. All consenting hospitalized patients who met the EM case definition and had CSF laboratory studies consistent with EM were enrolled in the study.

Patients were excluded if they 1) had a non-infectious etiology for their encephalitic syndrome, such as trauma, toxic exposures, cerebrovascular accident, or known malignancy; or 2) were unable to provide informed consent/assent without a legally authorized representative available.

### Epidemiological data

A questionnaire covering all enrolled acute EM patients was completed using their medical histories. This included demographics, clinical symptoms, and CSF laboratory parameters (WBC count, predominant leukocyte type, protein, and glucose). Patients, or their surrogates, were asked about exposure to ticks, dogs, and animal bites. Data were recorded in electronic questionnaires created and analyzed in Epi Info version 7. Statistical differences for categorical data were calculated using the chi-square or Fisher Exact tests; for continuous normally distributed data using the ANOVA test; and for data not normally distributed using the Kruskal Wallis test. In all cases, a p value <0.05 was considered significant. The 95% confidence intervals (CI) for a single proportion (cumulative incidence) were calculated using Wilson’s approximation for a binomial distribution in Epitools [27,28].

### Specimen collection

**Fig 1:**
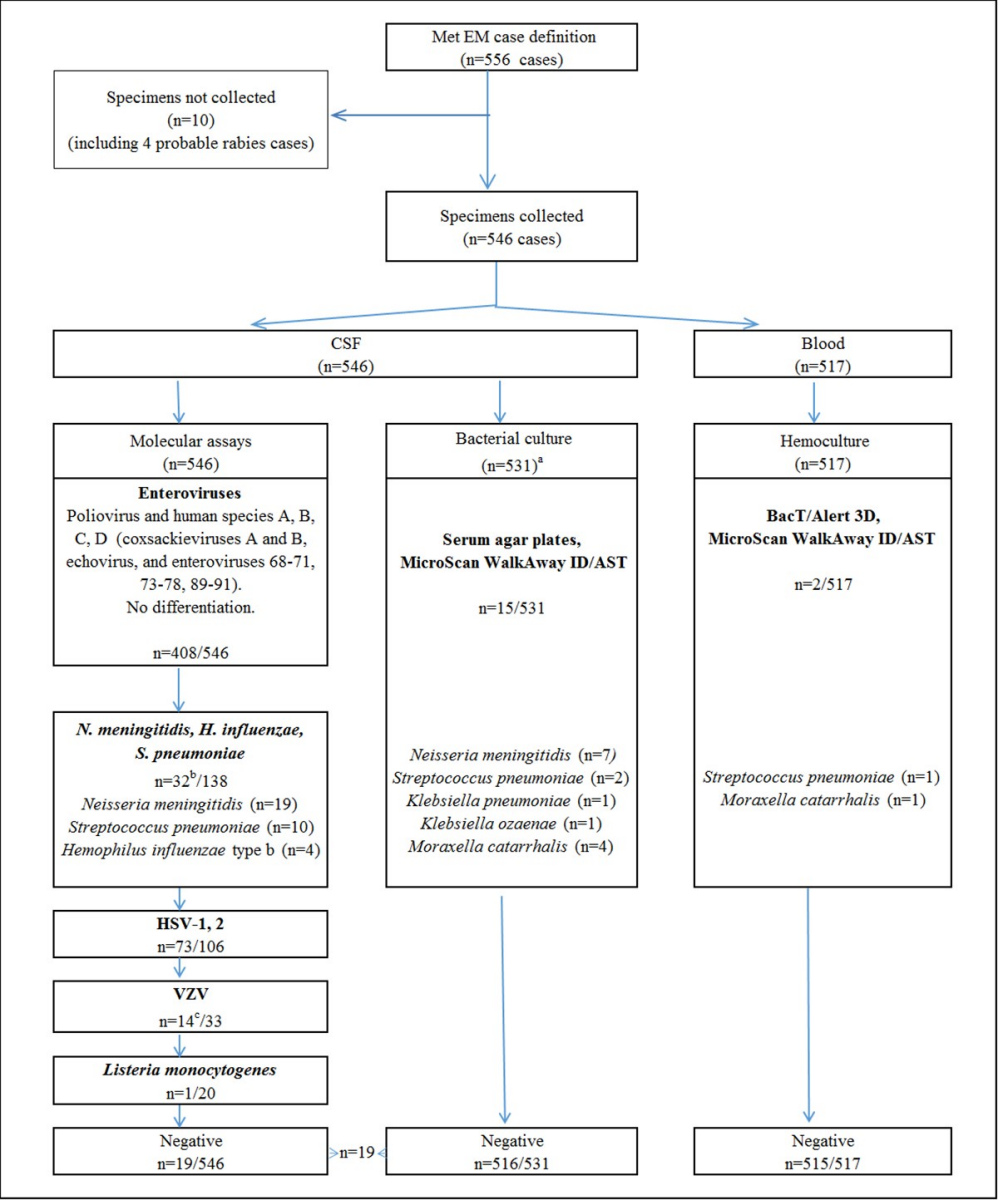
Flow chart for the diagnostic algorithm performed with associated test results. 546 CSF samples and 517 blood samples were taken from 556 patients. Out of 546 collected CSF samples, all 546 were tested in molecular assays and 331 were tested for bacterial culture. In molecular assays 494/546 samples were positive for viruses, 33/546 samples were positive for bacteria, and one sample was positive for both. 15/531 CSF samples and 2/517 blood samples were positive for bacterial culture. Nineteen CSF samples remained negative. ^a^15 cases with mild symptoms had less than 1ml cerebrospinal fluid collected and culture was not conducted. ^b^One sample was positive for both *N*. *meningitidis* and Hib. ^c^One sample was positive for both *N*. *meningitidis* (tested at the earlier step) and VZV.

As part of clinical care, samples of CSF (1–1.5 ml) and blood (2 ml from children <2 years old, 3 ml from older persons) were collected from each enrolled patient. All testing was performed at the Shymkent City Hospital laboratories. CSF and blood were divided into two portions: one each sent to the bacteriology laboratory for culture, and the second to the clinical diagnostic laboratory for molecular and cytology testing (Fig 1). Samples from probable rabies cases, defined as patients who had acute encephalitis with hyperactivity or paralytic syndromes resulting in death, and dog or another suspected rabid animal bite in the anamnesis, were not tested, but questionnaire data from these cases were included in the analyses [29].

### Laboratory procedures

#### Bacterial culture

CSF samples were inoculated onto chocolate and serum agar plates and incubated in an atmosphere of 5% CO_2_ using a candle-extinction jar or gas-generating packs at 35°C for 18–24 hours. Presumptive identification was based on growth characteristics, specific colony morphology, and Gram stain properties [30]. Isolated colonies were inoculated into the appropriate MicroScan WalkAway Combination Panels (Beckman Coulter, USA)—Pos. Breakpoint Combo 29, Neg. Breakpoint Combo 42 for identification and antimicrobial sensitivity testing (AST) and Haemophilus-Neisseria identification (HNID) panel for identification according to manufacturer’s instructions.

Blood specimens were cultured on the BacT/Alert 3D Microbial Detection System (bioMérieux, France) for up to 7 days, using the FA Aerobic or PF Pediatric bottles according to manufacturer’s instructions. Alarm positive bottles were sub-cultured on chocolate agar plates and incubated overnight at 35°C prior to Gram staining. Identification and AST were performed on the MicroScan WalkAway Combination Panels as described earlier.

All *N*. *meningitidis* positive cultures had further AST testing performed using disk diffusion methodology and results were interpreted according to Clinical and Laboratory Standards Institute guidelines 2015 [31,32].

#### Molecular assays

Nucleic acid extractions from CSF were performed using the AmpliSens RIBO-prep kit for RNA or the RIBO-sorb for DNA (InterLabServices, Moscow, Russian Federation) according to the manufacturer’s instructions. Extracts were aliquoted and frozen at -20°C until tested. Testing was performed according to the diagnostic algorithm detailed in Fig 1. Testing for EV was performed with the AmpliSens Enterovirus—FRT PCR kit. This kit detected poliovirus and enterovirus species А, В, С, D (coxsackieviruses A and В, echovirus, and EV 68–71, 73–78, 89–91 without differentiation. Negative samples were further tested by the AmpliSens *N*. *meningitidis*/*H*. *influenzae*/*S*. *pneumoniae—*FRT PCR kit; after which negative samples were tested using the AmpliSens HSV I, II and VZV—FRT PCR kits for herpes simplex and varicella-zoster viruses. Finally, all remaining negative samples were tested with the AmpliSens *Listeria monocytogenes* screen-titer—FRT PCR kit. All assays were run on a Rotor-Gene 6000 instrument (Corbett Life Science, Sydney, Australia) according to specific kit conditions.

A patient was considered positive for a pathogen if it was identified by either culture or PCR testing. We randomly selected 23 EV reverse transcriptase (RT)-PCR positive cases and sent both CSF and serum samples to the Kazakh Republican Laboratory for presumptive EV group identification. Virus cultivation from CSF samples used rhabdomyosarcoma (RD), and human embryonic kidney (HEp-2) cells [33,34]. Cultures with cytopathic effect were serotyped with EV diagnostic sera (echovirus types 6, 9, 11, 13, 25, 30 and coxsackievirus group B) (M.P. Chumakov Institute for Polio and Viral Encephalitis, Moscow, Russian Federation and Netherlands National Institute for Public Health and the Environment, Bilthoven, Netherlands). Serum specimens were tested in a neutralization assay for the presence of antibodies to echovirus 13 (E13).

## Results

Over the 12 months of surveillance, 556 cases of encephalitis and/or meningitis met our case definitions and were enrolled in the study. CSF samples for etiology testing were collected from 546 (98.2%) patients. Samples were not collected from ten patients–sampling from five patients were overlooked, one patient died before samples could be taken, and four patients were probable rabies cases for which CSF was not tested. Cultures were performed on 531/546 CSF samples (in 15 cases < 1ml CSF sample was collected hence no culture was performed) and 517 paired blood samples; PCR tests were performed on all 546 CSF samples (Fig 1).

### Incidence and case fatality rates

In this study, we calculated the overall incidence rate (IR) of EM was 19.2 per 100,000 persons; the incidence rate of EM with viral etiology (17.0 per 100,000 persons) was 13.3 times higher (p<10^−8^) than the incidence rate of EM of bacterial etiology (1.3 per 100,000 persons).

**Fig 2:**
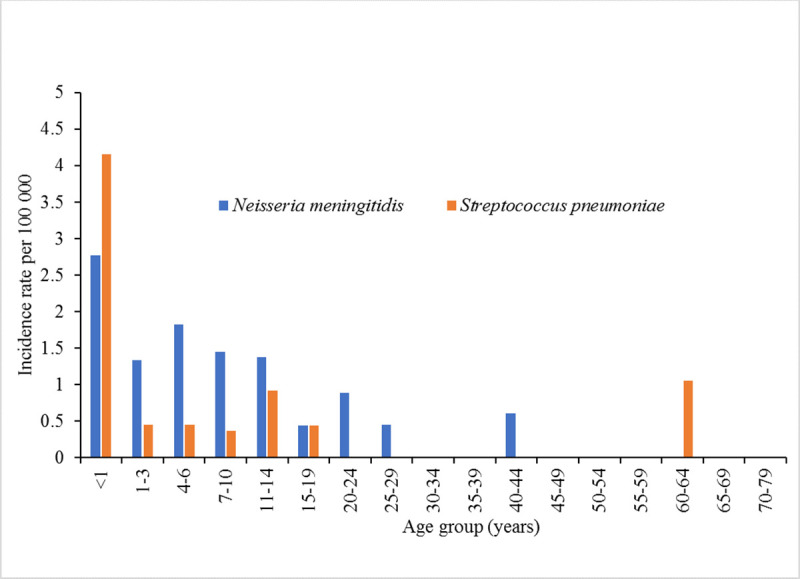
Incidence rates of meningococcal and pneumococcal EM by age group. Children <1-year-old had the highest IRs for bacterial EM; 4.2 and 2.8 per 100,000 children for pneumococcal and meningococcal EM, respectively. The IR of pneumococcal EM among children < 1 yr. of age was 8 times higher than the IR among children aged 1–14 years. No significant difference by age was observed for meningococcal EM.

Laboratory confirmation either by PCR or culture determined that the IR for EM caused by *N*. *meningitidis* and *S*. *pneumoniae* was 0.7 and 0.35 per 100,000 persons, respectively (Table 1). However, using culture results alone, as was the standard practice in Kazakhstan prior to this study, the IR for meningococcal EM was estimated to be 0.2 per 100,000 persons, three times lower (p = 0.008), and the IR for pneumococcal EM was estimated to be 0.07 per 100,000 persons five times lower (p = 0.02). Among children under 15 years old, IR of meningococcal and pneumococcal EM were 1.6 and 0.8 per 100,000 children, respectively, which are five (p = 0.0001) and eight (p = 0.005) times higher compared to individuals 15 years and older (Table 1). Children <1-year-old had the highest IRs for bacterial EM at 4.2 and 2.8 per 100,000 children for pneumococcal and meningococcal EM, respectively (Fig 2). The IR for pneumococcal EM among children < 1 yr. of age was 8.4 times higher than the IR among children aged 1–14 year (p = 0.02). No significant difference (p = 0.3) by these age groups of children was observed for meningococcal EM. No cases of EM caused by Hib, *Klebsiella*, or *Listeria* was identified among children <1 yr. of age (Table 2).

**Table 1:** Incidence rates of encephalitis/meningitis per 100,000 population by etiology in children and adults.

Etiology (number of cases*)	All cases	Children (<15years old)	Adults (≥15 years old)	p-value^3^
	IR^1^ (95% CI)^2^	IR (95% CI)	IR (95% CI)	
*Neisseria meningitidis* (n = 21)	0.7 (0.5–1.1)	1.6 (1–2.6)	0.3 (0.1–0.6)	0.0001
*Streptococcus pneumoniae* (n = 10)	0.3 (0.2–0.6)	0.8 (0.4–1.6)	0.1 (0.0–0.4)	0.005
*Hemophilus influenzae* type b (n = 3)	0.1 (0.0–0.4)	0.1 (0.0–0.6)	0.1 (0.0–0.4)	1.0
*Klebsiella* spp. (n = 2)	0.1 (0.0–0.2)	0 (0–0.4)	0.1 (0.0–0.4)	0.5
Enterovirus (n = 406)	14.0 (12.7–15.4)	35.9 (32.4–39.8)	2.3 (1.7–3.1)	<0.0001
Herpes simplex virus (n = 71)	2.4 (1.9–3.1)	4.9 (3.7–6.4)	1.2 (0.7–1.7)	<0.0001
Varicella-zoster virus (n = 13)	0.4 (0.3–0.8)	1 (0.5–1.8)	0.2 (0.1–0.5)	0.002

* Each patient is included only once in case count.

^1^IR- Estimated incidence rate per 100000.

^2^CI- Confidence interval.

^3^p-value compares between children <15 years old and adults ≥15 years old.

**Table 2:** Incidence rates of encephalitis/meningitis per 100,000 population by etiology in children <1 year old and (1–14) years old.

Etiology (number of cases*)	Children (<1 year old)	Children (1–14 years old)	RR^3^	p-value^4^
	IR^1^ (95% CI)^2^	IR^1^ (95% CI)^2^		
*Neisseria meningitidis* (n = 16)	2.8 (0.8–10.1)	1.5 (0.9–2.5)	1.8	0.3
*Streptococcus pneumoniae* (n = 8)	4.2 (1.4–12.2)	0.5 (0.2–1.2)	8.4	0.02
*Hemophilus influenzae* type b (n = 1)	0 (0.0–5.3)	0.1 (0.0–0.6)	U^5^	0.9
*Klebsiella* spp. (n = 0)	0 (0.0–5.3)	0 (0–0.4)	U^5^	0.9
*Listeria* (n = 1)	0 (0.0–5.3)	0.1 (0.0–0.6)	U^5^	0.9
*Enterovirus* (n = 362)	18.0 (10.5–30.8)	37.3 (33.5–41.4)	0.5	0.003
*Herpes simplex virus* (n = 49)	5.5 (2.2–14.2)	4.8 (3.6–6.4)	1.1	0.5
*Varicella-zoster virus* (n = 10)	0 (0.0–5.3)	1.1 (0.6–2.0)	U^5^	0.5
*Rabies* (n = 1)	0 (0.0–5.3)	0.1 (0.0–0.6)	U^5^	0.9

* Each patient is included only once in case count.

^1^IR- Estimated incidence rate per 100000.

^2^CI- Confidence interval.

^3^RR-risk ratio, comparing of incidence rates of EM between children <1 year old and (1–14) years old.

^4^p-value compares between children < 1 year old and children (1–14) years old.

^5^U-undefined.

The EV-associated EM IR was 14.0 per 100,000 persons. For children less than 15 years old, the IR was 35.9 per 100,000 children, 15.6 times higher (p<0.0001) than among adults (Table 1).

For children <1 yr. of age, the highest rates of EM were for EV (18.0 per 100,000 children) and HSV (5.5 per 100,000 children); rabies and VZV cases were not identified (Table 2).

Using PS/DCI and school enrollment population data, the estimated IR of EV-associated EM for 0–6 years old enrolled in PS/DCI (40.8 per 100,000 children) was 2.1 times higher (p<10^−5^), compared to those who were not enrolled in PS/DCI (19.1 per 100,000 children). For school pupils aged 6–17 years old, the rate was 39.3 per 100,000 persons; during the school year the IR was 22,1 per 100,000 children.

Twelve of the 556 cases died (CFR = 2.2%), including nine cases with known etiology (one each associated with *N*. *meningitidis*, EV, VZV, HSV, and *L*. *monocytogenes*, and four probable rabies cases) (Table A in S1 Appendix).

### Etiology of EM

An etiological agent was identified in 531/556 (95.5%) cases, with 4.5% (25/556) of unknown etiology including the six non-tested cases (Table 3).

**Table 3:** Acute encephalitis/meningitis etiology by clinical presentation of infection (n = 556).

Etiology	All cases (n = 556)	Children (age < 15), (n = 467)	Adults (age ≥ 15), (n = 89)	Meningitis (n = 510)	Meningo-encephalitis (n = 35)	Encephalitis (n = 11)
	n (%)	n (%)	n (%)	n (%)	n (%)	n (%)
**Viral, Total:**	494 (88.8)	422 (90.4)	72 (80.9)	466 (91.4)	20 (57.1)	8 (72.7)
Enterovirus	406^a^ (73.0)	362 (77.5)	44 (49.4)	396 (77.6)	8 (22.9)	2 (18.2)
Herpes simplex virus1/2	71^b^ (12.8)	49 (10.5)	22 (24.7)	58 (11.4)	11 (31.4)	2 (18.2)
Varicella-zoster virus	13 (2.3)	10 (2.1)	3 (3.4)	12 (2.4)	1 (2.9)	0 (0)
Rabies virus	4^c^ (0.7)	1 (0.2)	3 (3.4)	0 (0)	0 (0)	4 (36.4)
**Bacterial, Total:**	37 (6.7)	26 (5.6)	11 (12.4)	25 (4.9)	10 (28.6)	2 (18.2)
*Neisseria meningitidis*	21^d^ (3.8)	16 (3.4)	5 (6.4)	13 (2.5)	7 (20)	1 (9.1)
*Streptococcus pneumoniae*	10 (1.8)	8 (1.7)	2 (2.2)	7 (1.4)	3 (8.6)	0 (0)
*Klebsiella pneumoniae*	1^e^ (0.2)	0 (0)	1 (1.1)	1 (0.2)	0 (0)	0 (0)
*Klebsiella ozaenae*	1^f^ (0.2)	0 90)	1 (1.1)	1 (0.2)	0 (0)	0 (0)
*Hemophilus influenza* type b	3 (0.5)	1 (0.2)	2 (2.2)	3 (0.6)	0 (0)	0 (0)
*Listeria monocytogenes*	1 (0.2)	1 (0.2)	0 (0)	0 (0)	0 (0)	1 (9.1)
**Known etiology, Total:**	531 (95.5)	448 (95.9)	83 (93.2)	491 (96.3)	30 (85.7)	10 (90.9)
Negative	19 (3.4)	15 (3.2)	4 (4.5)	15 (2.9)	3 (8.6)	1 (9.1)
Not tested	6 (1.1)	4 (0.9)	2 (2.2)	4 (0.8)	2 (5.7)	0 (0)
**Unknown etiology, Total:**	25 (4.5)	29 (4.1)	6 (6.7)	19 (3.7)	5 (14.3)	1 (9.1)
Total:	556 (100)	467 (100)	89 (100)	510 (100)	35 (100)	11 (100)

Each patient is only included in a case count once.

^a^Included coinfection with *M*. *catarrhalis* (n = 3*)*.

^b^Included coinfection *M*. *catarrhalis* (n = 2).

^c^Based on clinical and epidemiology evidence (not tested).

^d^Included as *N*. *meningitidis* when coinfections identified with enterovirus (n = 2), Varicella-zoster virus (n = 1), *H*. *influenza* type b (n = 1).

^e^Included as *K*. *pneumoniae* when coinfection identified with herpes simplex viruses (n = 1).

^f^Included as *K*. *ozaenae* when coinfection identified with herpes simplex viruses (n = 1).

Among cases, the prevalence of viral EM was 88.8% (494/556), with 73.0% (406/556) of probable etiology attributed to EVs; 12.8% (71/556) to HSV, and 2.3% (13/556) to VZV (Table 3). Rabies was diagnosed in 0.7% (4/556) of cases based on history of dog bite, clinical symptoms, and fatal outcome.

EV serotyping was performed on 23 randomly selected RT-PCR-positive CSF samples. We detected E13 in 73.9% (17/23) samples. The remaining six samples could not be identified with the available sera. In the virus culture, E13 was isolated in 52.2% (12/23) of the CSF samples. Serum neutralization test was performed on 15 serum samples paired with CSF samples, E13 neutralizing antibodies were detected in 66.7% (10/15) samples, titers ranged between 1:2 to 1:16. Five paired samples, CSF and serum, were positive by both assays.

The case prevalence for bacterial EM was 6.7% (37/556). Meningococcal etiology amounted to 3.8% (21/556) of all cases and 56.7% of bacterial EM cases (14 positives by PCR only, two positives by culture only, and five positives by both PCR and culture), (Table 3). Three meningococcal serogroups were identified on culture positive cases: 43% (3/7) were group A, 28.5% (2/7) were group B, and 28.5% (2/7) were group C. *S*. *pneumoniae* was isolated in 27.0% (10/37) cases including eight positives by PCR only and two positives by both culture and PCR. *Hemophilus influenzae* type b (Hib) was isolated in 8.1% (3/37) of cases by PCR only. Two cases of *Klebsiella* spp. (5.4%) including *K*. *pneumoniae* and *K*. *ozaenae* were identified by culture, and one case (2.7%) of *L*. *monocytogenes* was identified by PCR.

Data on antibacterial medicines (AB) use was available only during the period of hospitalization. AB use prior to CSF collection was similar between PCR positive cases which were negative for culture (20.8% (5/24)) and culture positive bacterial etiology cases (18.2% (2/11)).

In tabulating coinfections, etiologies were only counted once, so the 4 *Moraxella catarrhalis* identifications with EV (n = 3) and HSV (n = 1) coinfections were reported as the respective viral etiologies. HSV was identified as a coinfecting pathogen both times *Klebsiella* spp. were identified in CSF culture; these were counted as bacterial etiology. There were 4 cases of co-infections with *N*. *meningitidis*, EV (n = 2) and a single case each with VZV and Hib. We considered this an indicative of *N*. *meningitidis* infection.

We classified cases by characteristics of meningitis, encephalitis, or meningoencephalitis. Most cases (91.7%; 510/556) had clinical characteristics of meningitis, while 2.0% (11) met the case definition for encephalitis, and 6.3% (35) had a meningoencephalitic appearance (Table 3). Viral etiology predominated in all three groups, with the largest proportion among meningitis (91.4%; 466/510) and smallest among meningoencephalitis (57.1%; 20/35) (Table 3).

**Fig 3:**
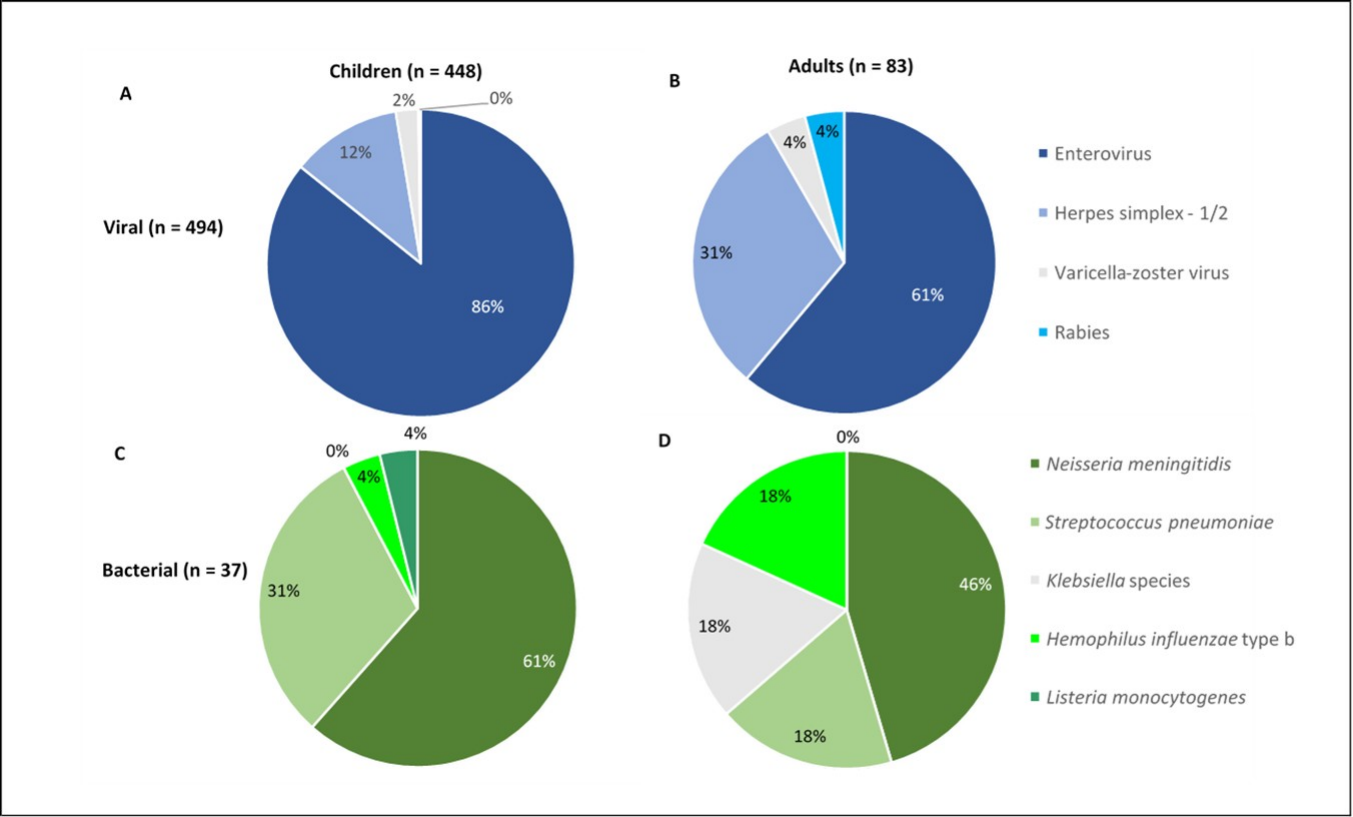
Viral and bacterial etiologies of acute encephalitis/meningitis in adults and children. (A) In children (< 15 years old) the largest proportion of EM was caused by enteroviruses (86%; 362/422), herpes simplex viruses (12%; 49/422), varicella-zoster virus (2%; 10/422) and probable rabies (0.2%; 1/422). (B) For adults (≥ 15 years old), the proportion of enterovirus cases was lower at 61% (44/72), and herpes simplex virus infections higher at 31% (22/72). (C) For bacterial etiologies in children, the largest proportion of cases was caused by *N*. *meningitidis* at 61% (16/26), followed by *S*. *pneumoniae* at 31% (8/26). (D) In adults, bacterial etiology due to *N*. *meningitidis* was lower than children but remained the largest bacterial etiologic agent at 46% (5/11).

In children under 15 years old, the largest proportion of EM was caused by EV (85.8%; 362/422), followed by HSV (11.6%; 49/422) and VZV (2.4%; 10/422) (Fig 3A). Among adults, the proportion of EV cases was lower at 61.1% (44/72) and HSV infections higher at 30.6% (22/72) (Fig 3B). The most common bacterial etiologies identified in children predominantly were *N*. *meningitidis* (61.5%; 16/26), followed by *S*. *pneumoniae* (30.8%; 8/26) (Fig 3C). In adults, bacterial etiology due to *N*. *meningitidis* was lower than children but remained the largest bacterial etiologic agent at 45.5% (5/11) (Fig 3D). In children under 1 year old, the largest proportion of EM was caused by EV (59,1%; 13/22), followed by HSV (18.2%; 4/22), *S*. *pneumoniae* (13,6%; 3/22) and *N*. *meningitidis* (9,1%, 2/22).

### Case characteristics

We compared clinical signs and symptoms between cases with bacterial and viral meningitis (Table B in S1 Appendix). Cases of meningitis with bacterial etiology were 10 times more likely to have rash (p<0.005), 3.3 times more likely to have Kernig’s and Brudzinski’s signs (p<0.001), and more likely to have a higher average body temperature at the disease onset (p = 0.001) than cases with viral etiology. In contrast, vesicular pharyngitis was 2.6 times more common in viral meningitis (p<0.001). There were no statistical differences (p>0.05) between bacterial and viral meningitis by age or gender, and no differences observed between bacterial or viral encephalitis and meningoencephalitis cases by age, gender, or clinical signs (Table B in S1 Appendix). A further comparison of CSF laboratory results among meningitis cases (Table B in S1 Appendix) showed the median leukocyte count for bacterial meningitis was 563 x 10^6^/L, four times higher than for viral meningitis (139 x 10^6^/L). This statistically significant difference (p<0.004) may have limited clinical diagnostic relevance since the proportion of cases with low (< 100 x 10^6^/L) leukocytosis was similar (p = 0.25) between bacterial (28%; 7/25) and viral (39.5%; 184/466) meningitis; only 40% (10/25) of bacterial meningitis had leukocytosis ≥ 1000 x 10^6^/L. The predominate cells for bacterial meningitis were neutrophils (65%) versus lymphocytes (70%) in viral meningitis, and the average protein concentration was twice as high in bacterial versus viral meningitis (1.4 vs. 0.6 g/L respectively). There were no differences in glucose levels between viral and bacterial meningitis (data available for 68% (17/25) of bacterial meningitis cases) (Table A in S1 Appendix).

### Case distribution by time

**Fig 4:**
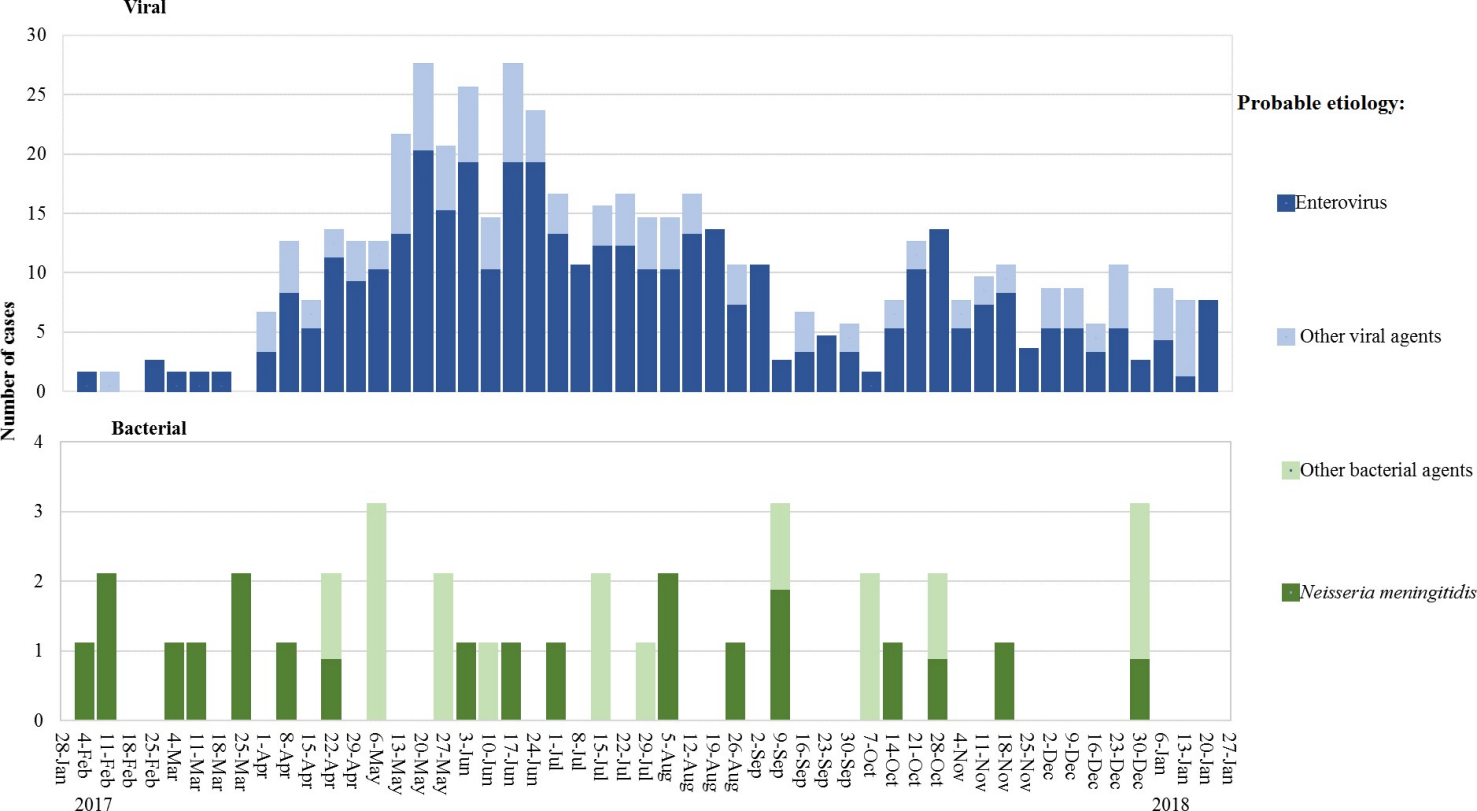
Epidemiological curves of laboratory-confirmed acute encephalitis/meningitis in South Kazakhstan oblast, February 2017—January 2018. The viral etiology epidemiological curve shows an increase in enterovirus cases in March 2017 that peaked the first week of May and stayed high until the end of June. Cases then gradually decreased until early October. A second wave starting the first week of October was observed, peaking at the end of the month at a level half that of the first upsurge. Meningococcal meningitis cases were identified throughout the whole year.

The viral etiology epidemiological curve (Fig 4) shows an increase in EV cases in March 2017 that peaked the first week of May and remained elevated until the end of June (20–23 cases a week). Cases then gradually decreased until early October, when 1–2 cases were registered per week. A second wave starting the first week of October was observed, peaking at the end of the month at a level half that of the first upsurge. Meningococcal meningitis cases were identified in SKO throughout the whole year (Fig 4).

### Treatment of cases

Antibacterial medicines (AB) were prescribed in 97.2% (480/494) of virus-associated EM cases and all bacterial-associated cases. The average duration of treatment was 8.5 days and ranged from one to 23 days. There were no differences in treatment duration between viral- and bacterial-associated EM (Table C in S1 Appendix).

Antibacterial resistance was high in our samples. In our study, we found that 50% of *N*. *meningitidis* isolates were resistant to gentamycin and ciprofloxacin, 43% resistant to penicillin, and 20% resistant to ceftriaxone. (Table D in S1 Appendix).

## Discussion

Our study is the first systematic surveillance of EM conducted in SKO, a region where little was known about the incidence and etiology of EM. We calculated the overall incidence of EM at 19.2 cases per 100,000 persons, which is similar to rates reported from China [35].

In our study, pathogens were identified in 95.5% of cases. In other studies, the identification of causative agents was lower, typically ranging from 58–66% [6,23]. However, studies demonstrating high rates of identified pathogens have been reported. In Finland, surveillance of an outbreak of aseptic meningitis confirmed enteroviral origin in 80% of cases [36]. During a summer outbreak of EV meningitis in Switzerland, E30 was the causative agent in 85% of aseptic meningitis cases confirmed by RT-PCR [10]. In Egypt, which is endemic for streptococcal, meningococcal, and Hib meningitis, 90% positivity rate for suspected bacterial meningitis cases with culture negative CSF was confirmed by RT- PCR testing [37].

The majority (88.8%) of cases were of viral etiology, and while it is common for EV to comprise the largest etiological fraction, our detection of 73% was higher than among countries, such as UK and Finland (26–55%) [5,8]. Our high rate of positivity could be explained by hyper endemicity of EV, or the potential occurrence of a seasonal outbreak. The lack of baseline data makes it difficult to confirm the assumption. Additional years of surveillance are currently under way, to help resolve this question.

Most EV cases occurred in summer among children aged 4–14 years old, which is consistent with EV seasonality and typical age distribution [12,38–40]. We identified the highest IR of EV EM for children ≤ 6 years old attending PS/DCI (40.8 per 100,000 children), which is 2.1 times higher than the rate in the same age group who stayed at home. This finding indicates that enrollment in preschool or day care could be a risk factor for EV EM [41]. Insufficient sanitary-hygienic conditions at institutions could possibly contribute to person-to person transmission of EV. These institutions could also be the source of EV in adult family members [42]. This should be considered for epidemiological anamnesis collection and recommendations made to improve the sanitary-hygienic regimen including proper and frequent handwashing.

We identified a bacterial agent in 6.7% EM cases. The three most common bacterial agents of EM were *N*. *meningitidis*, *S*. *pneumoniae* and Hib. They are also the leading cause of bacterial meningitis in other countries [6,43]. The IR for bacterial pathogens using both PCR and culture results were statistically 3–5 times higher compared to culture-only results. This illustrates that without PCR based molecular testing, rates of bacterial EM are underestimated. AB were prescribed for treatment in nearly all EM cases, even though the majority of cases had viral etiology. Many studies comparing bacterial culture to PCR as a diagnostic tool show the latter has greater sensitivity (1.7 to 3.1 times) and shorter turn-around times (3–4 hours compared to at least 48 hours) and that AB usage prior to CSF sample collection decreases the sensitivity of culture but not PCR [44–46]. In Kazakhstan AB are easily accessed by the population as they can be purchased without a prescription. Also, usage data prior to hospitalization is not obtained. From our incomplete data no differences were noted for AB started before CSF collection between PCR and culture methods (20.8% and 18.2% respectively).

Testing for this project could not be used for clinical decision making for several reasons: testing was not performed on-site; molecular assays were batched because of funding restraints and reagent stockouts were experienced because of higher than expected testing demand. However, the results for this surveillance will be used to provide recommendations to the appropriate authorities to change current guidelines.

Vaccination against bacterial meningitis is an effective strategy for reduction of cases and therefore reduction of antibiotics usage. In England and Wales, the rate of bacterial meningitis decreased significantly after the introduction of the conjugate Hib vaccine for infants [43]. Meningococcal and pneumococcal vaccines also contributed to a considerable decline in bacterial meningitis in New Zealand and in the United States [47,48]. In SKO, immunization against Hib and *S*. *pneumoniae* were introduced in 2008 and 2014 respectively, with coverage of children 1 year or less at 95.9% and 98.4%, respectively (based on unpublished country immunization report, 2017); however, their effectiveness cannot be determined, as pre-immunization surveillance data are not available. Our study shows the rates of pneumococcal EM are comparable to the USA for children after implementation of vaccination [48]. Furthermore, we only identified one case of Hib in children, which may suggest a successful immunization programme. In Kazakhstan, vaccination against meningococcus has not yet been implemented, so the serogroup information generated from the culture positive isolates is timely and relevant. It will be important to type the remaining PCR positive samples.

Coinfection of EV was detected with *N*. *meningitidis* and *M*. *catarrhalis* in 1.2% of cases. Previous studies have reported the similar proportions of 2.8% and 1.3% of bacterial agents from patients with EV meningitis. These studies also identified coinfection of EV with *N*. *meningitidis*, *Salmonella*, *S*. *pneumonia*, Hib and *S*. *aureus* [49,50]. In our study, we identified three unusual coinfections of HSV with *K*. *pneumonia* and *K*. *ozaenae* in two cases, and Hib with *N*. *meningitidis* in one case. In these cases, the HSV and Hib were identified by PCR and *K*. *pneumonia*, *K*. *ozaenae*, and *N*. *meningitidis* by culture.

We could not identify an etiology for 4.5% of cases. Further studies should include testing for paramyxoviruses (mumps and measles viruses), other herpesviruses (not only HSV1,2 and VZV), Flaviviridae (TBEV) and possibly adenoviruses as has been reported in the literature [1]. TBEV in the SKO is not considered endemic, so we did not include this in our diagnostic algorithm, but a study in 2016 [51] showed presence of TBEV in ticks in the southern region of Kazakhstan. This provides justification for including testing for TBEV in the future surveillance activities for EM.

A comparison of bacterial and viral associated meningitis cases demonstrated the expected differences in CSF laboratory characteristics: a higher proportion of neutrophils (65% vs 30%) and protein levels (1.4 g/L vs 0.9 g/L) in bacterial cases compared to viral, and a higher lymphocyte proportions of 70% in viral to 35% in bacterial cases. However, only 40% of bacterial meningitis cases had typically high leucocyte counts (≥1000 x10^6^/L) in CSF [52]. Of note, CSF glucose levels in our investigation did not differ between bacterial and viral meningitis. This is curious, as bacterial meningitis is generally associated with a low CSF glucose level. It is possible that in early stages of disease, the CSF glucose levels were normal, or perhaps bacterial/viral coinfections were not detected as not all samples had all diagnostic assays. Serum glucose levels were not available for all cases, but most cases were young and healthy, so presumptively normoglycemic. No other organ abnormalities were mentioned by caring physicians in the study population. Despite the differences of some clinical symptoms/signs of meningitis between the two etiologies including vesicular pharyngitis, rash, Kernig’s and Brudzinski’s signs the considerable overlap in other symptoms/signs makes distinguishing the two etiologies challenging without appropriate laboratory diagnostics. Similarities in clinical signs explains the high level of antibacterial administration to cases with a viral etiology. This study indicates that possible changes to the algorithm should consider not prescribing AB for children, especially in the summer period, as most cases during this period are viral. Molecular testing to determine etiology needs to have short turn-around times so results can be used for clinical management of patients.

There are several limitations in our study. There is possible selection bias as it is likely that there were cases that were not hospitalized, so were not captured in this study. Further, we have not captured data from possible cases that died before hospitalization, resulting in possible underestimation of IR and CFR. Extensive typing of viruses or bacteria was not performed; this could provide valuable information for vaccine introduction. Information on co-infections was limited as not all samples were tested by all assays. We studied EM in one area, SKO, of Kazakhstan and it is likely not indicative of the entire country.

The investigation covered a single year, which prevents comparison of trends over time. Meningitis outbreaks in Kazakhstan vary over time. There have been years in which no outbreaks occurred, while in some years large EM outbreaks have been documented. Continual EM surveillance is needed to document the patterns of EM incidence.

Identifying the etiology of EM in Kazakhstan will assist the country in developing appropriate recommendations for hygiene and sanitation improvements, immunization programme assessments to guide appropriate meningococcal vaccination strategies, and in the shorter term avoid unnecessary antibacterial treatment which could help in combating the drug resistance menace. Expanding the laboratory diagnostic assays to include PCR for both bacterial and viral pathogens known to cause encephalitis and meningitis will aid in further understanding the etiology and provide the true incidence of disease, allowing for initiating and implementing suitable public health measures and appropriate treatments for EM patients. Complete typing of enteroviruses, beyond pan-viral assays, will allow for identification of specific subtypes, further directing appropriate public health actions. This study clearly demonstrates the importance of updating and implementing laboratory diagnostic testing strategy to better serve the people of Kazakhstan.

## Supporting information

S1 Table Dataset. (XLSX)

S1 Appendix This appendix contains Tables A, B, C and D. Table A. Case fatality rates among acute encephalitis, meningoencephalitis and meningitis patient. Table B. Characteristics of acute encephalitis, meningitis, and meningoencephalitis cases by bacterial or viral etiology (n = 531). Table C. Use of antibacterial and antiviral treatment for acute encephalitis or meningitis patients. Table D. Susceptibility of *Neisseria meningitidis* (n = 7) isolated from cerebrospinal fluid to antibacterial medicines.
(PDF)

S2 Appendix Questionnaire in English. (PDF)

S3 Appendix Questionnaire in Russian. (PDF)

S1 Text Abstract in Russian. (PDF)
