# Supplementary material for: Etiology of acute meningitis and encephalitis from hospital-based surveillance in South Kazakhstan oblast, February 2017—January 2018
Source: PLoS One. 2021 May 14;16(5):e0251494. doi: 10.1371/journal.pone.0251494 (PMC8121361; doi:10.1371/journal.pone.0251494)
Supplement: S1 Appendix — This appendix contains Tables A, B, C and D. Table A. Case fatality rates among acute encephalitis, meningoencephalitis and meningitis patient. Table B. Characteristics of acute encephalitis, meningitis, and meningoencephalitis cases by bacterial or viral etiology (n = 531). Table C. Use of antibacterial and antiviral treatment for acute encephalitis or meningitis patients. Table D. Susceptibility of Neisseria meningitidis (n = 7) isolated from cerebrospinal fluid to antibacterial medicines. (PDF) [file pone.0251494.s002.pdf]

**S1 Appendix**

**Table A. Case fatality rates among acute encephalitis, meningoencephalitis and meningitis patients.**

**Table B. Characteristics of acute encephalitis, meningitis, and meningoencephalitis cases by bacterial or viral etiology (n=531).**

**Table C. Use of antibacterial and antiviral treatment for acute encephalitis or meningitis patients.**

**Table D. Susceptibility of *Neisseria meningitidis* (n=7) isolated from cerebrospinal fluid to antibacterial medicines.**

**Table A. Case fatality rates among acute encephalitis, meningoencephalitis and meningitis patients.**

| **Etiology** | **Lethal cases** | | | | **Number of cases** | **Case fatality rate (%)** |
|---|---|---|---|---|---|---|
| | **Total Number** | **Encephalitis** | **Meningo-encephalitis** | **Meningitis** | | |
| **Viral, Total:** | **7** | **5** | **2** | **0** | **494** | **1.4** |
| Rabies virus | 4 | 4 | 0 | 0 | 4 | 100 |
| Enterovirus | 1 | 0 | 1 | 0 | 406 | 0.2 |
| Herpes simplex virus 1/2 | 1 | 1 | 0 | 0 | 71 | 1.4 |
| Varicella-zoster virus | 1 | 0 | 1 | 0 | 13 | 7.7 |
| **Bacterial, Total:** | **2** | **1** | **1** | **0** | **37** | **5.4** |
| *Neisseria meningitidis** | 1 | 0 | 1 | 0 | 21 | 4.8 |
| *Listeria monocytogenes* | 1 | 1 | 0 | 0 | 1 | 100 |
| *Streptococcus pneumoniae* | 0 | 0 | 0 | 0 | 10 | 0 |
| *Klebsiella pneumonia* | 0 | 0 | 0 | 0 | 1 | 0 |
| *Klebsiella ozaenae* | 0 | 0 | 0 | 0 | 1 | 0 |
| *Haemophilus influenza*e type b | 0 | 0 | 0 | 0 | 3 | 0 |

| | | | | | | |
|---|---|---|---|---|---|---|
| Not tested | 2 | 0 | 2 | 0 | 6 | 33.3 |
| Negative | 1 | 1 | 0 | 0 | 19 | 5.3 |
| **Total** | **12** | **7** | **5** | **0** | **556** | **2.1** |
| **Clinical type:** | | | | | | |
| Encephalitis | **7** | | | | **11** | **63.6** |
| Meningoencephalitis | **5** | | | | **35** | **14.3** |
| Meningitis | **0** | | | | **510** | **0** |

* *N. meningitidis* + VZV (n=1)

**Table B. Characteristics of acute encephalitis, meningitis, and meningoencephalitis cases by bacterial or viral etiology (n=531).**

| Characteristics | Meningitis | | | | Encephalitis | | | | Meningoencephalitis | | | |
|---|---|---|---|---|---|---|---|---|---|---|---|---|
| | Viral (n=466) | Bacterial (n=25) | RR[1] | P - value[2] | Viral (n=8) | Bacterial (n=2) | RR[1] | P - value[2] | Viral (n=20) | Bacterial (n=10) | RR[1] | P - value[2] |
| | n (%) | n (%) | | | n (%) | n (%) | | | n (%) | n (%) | | |
| **Demographics** | | | | | | | | | | | | |
| Sex, Male | 256 (54.9%) | 18 (72.0%) | 0.7 | 0.8 | 5 (62.5%) | 2 (100%) | 0.6 | 1 | 13 (65.0%) | 5 (50.0%) | 1.3 | 0.3 |
| Age (years) [3] | 9.9 (0.2-55.8) | 16.2 (0.4-64.4) | | 0.1 | 21.3 (0.2-62.5) | 1.4 (0.6-2.2) | | 0.4 | 7.1 (0.3 -25.0) | 10.4 (0.3-40.2) | | 1 |
| **Clinical signs** | | | | | | | | | | | | |
| Body temperature (°C)[3] | 38.4 (37-40) | 38.8 (37.6-40) | | 0.001 | 38.6 (37-40) | 37.5 (37-38) | | 0.2 | 38.7 (38-40) | 38.6 (37.5-40.0) | | 0.7 |
| Headache | 449 (96.3%) | 22 (88.0%) | 1.1 | 0.4 | 4 (50%) | 1 (50%) | 1 | 1 | 13 (65.0%) | 7 (70.0%) | 0.9 | 1 |
| Neck stiffness | 439 (94.2%) | 22 (88.0%) | 1.1 | 0.2 | 0 | 0 | 0 | | 19 (95.0%) | 10 (100%) | 0.6 | 1 |
| Vomiting | 447 (95.9%) | 23 (92.0%) | 1.05 | 0.3 | 5 (62.5%) | 1 (50%) | 1.1 | 1 | 15 (75.0%) | 7 (70.0%) | 1.1 | 1 |

| | | | | | | | | | | | | |
|---|---|---|---|---|---|---|---|---|---|---|---|---|
| Brudzinski's sign | 86 (18.4%) | 15 (60.0%) | 0.3 | <0.001 | 0 | 0 | 0 | | 6 (30.0%) | 4 (40.0%) | 0.7 | 0.7 |
| Kernig's sign | 87 (18.7%) | 15 (60.0%) | 0.3 | <0.001 | 0 | 0 | 0 | | 6 (30.0%) | 4 (40.0%) | 0.7 | 0.7 |
| Convulsions | 0 | 0 | 0 | | 5 (62.5%) | 2 (100%) | 0.6 | 1 | 12 (60.0%) | 6 (60.0%) | 1 | 1 |
| Rash | 5 (1.1%) | 3 (12.0%) | 0.1 | 0.005 | 0 | 1 (50%) | U[4] | 0.2 | 1 (5.0%) | 1 (10.0%) | 0.5 | 1 |
| Coma | 0 | 0 | 0 | | 4 (50%) | 1 (50%) | 1 | 1 | 4 (20.0%) | 3 (30%) | 0.7 | 0.3 |
| Vesicular pharyngitis | 288 (61.8%) | 6 (24.0%) | 2.6 | <0.001 | 1 (12.5%) | 0 | U[4] | 1 | 4 (20.0%) | 0 | U[4] | 1 |
| Excitation | 3 (0.6%) | 0 | U[4] | 0.7 | 5 (62.5%) | 0 | U[4] | 0.4 | 1 (5.0%) | 3 (30.0%) | 0.2 | 0.1 |
| Paresis | 0 | 0 | 0 | | 3 (37.5%) | 0 | U[4] | 1 | 3 (15.0%) | 2 (20.0%) | 0.7 | 1 |
| Lurch | 0 | 0 | 0 | | 1 (12.5%) | 0 | U[4] | 1 | 3 (15.0%) | 1 (10.0%) | 1.5 | 1 |
| Tonsillitis | 42 (9.0%) | 1 (4.0%) | 2.2 | 0.7 | 0 | 1 (50%) | U[4] | 0.2 | 1 (5.0%) | 1 (10.0%) | 0.5 | 1 |
| Diarrhea | 25 (5.4%) | 0 | U[4] | 0.2 | 2 (25%) | 0 | U[4] | 1 | 2 (10.0%) | 0 | U[4] | 0.5 |
| Confusion | 0 | 0 | 0 | | 1 | 0 | U[4] | 1 | 1 | 0 | U[4] | 1 |

| | | | | | | | | | | | | |
|---|---|---|---|---|---|---|---|---|---|---|---|---|
| | | | | | (12.5%) | | | | (5.0%) | | | |
| Photophobia | 14 (3.0%) | 1 (4.0%) | 0.8 | 0.5 | 3 37.5%) | 0 | U[4] | 1 | 1 (5.0%) | 0 | U[4] | 1 |
| Lethargy | 0 | 0 | 0 | | 4 (50%) | 0 | U[4] | 0.5 | 5 (25.0%) | 1 (10.0%) | 2.5 | 0.6 |
| Bulging fontanelle | 3 (0.64%) | 1 (4.0%) | 0.2 | 0.07 | 0 | 0 | 0 | | 1 (5.0%) | 1 (10.0%) | 0.5 | 1 |
| **Cerebrospinal fluid (CSF) Analysis** | | | | | | | | | | | | |
| Leucocyte count ($x10^6$/L)[5] | 139 (58-378) | 563 (92-3,584) | | 0.004 | 146 (7-311) | 61 (27-96) | | 0.5 | 96 (42-228) | 168 (23-1066) | | 0.5 |
| Leucocyte count: | | | | | | | | | | | | |
| ≥1000 ($x10^6$/L) | 15 (3.2%) | 10 (40%) | | <0.001 | 0 | 0 | | | 3 (15%) | 3 (30%) | | 0.3 |
| <100 ($x10^6$/L) | 184 (39.5%) | 7 (28%) | | 0.25 | 2 (25%) | 2 (100%) | | 0.07 | 10 (50%) | 3 (30%) | | 0.2 |
| Neutrophils (%)[5*] | 30 (18-46) | 65 (22-80) | | 0.002 | 22 (20-24) | 18 | | 0.2 | 20 (12-32) | 60 (10-75) | | 0.1 |
| Lymphocytes (%)[5*] | 70 (53-82) | 35 (18-88) | | 0.002 | 78 (76-80) | 79 | | 0.6 | 80 (68-88) | 40 (25-90) | | 0.2 |
| Glucose (mmol/L)[5**] | 3.1 (2.9-3.4) | 3.2 (3.1-3.7) | | 0.1 | 2.9 (1.8-3.8) | 4.1 (3.5-4.6) | | 0.2 | 3.1 (2.9-3.5) | 2.9 (2.5-3.7) | | 0.3 |
| Protein (g/L)[3] | 0.6 (0.3-6.7) | 1.4 (0.3-5.9) | | 0.0003 | 0.6 (0.3-0.7) | 0.3 (0.3-0.3) | | 0.2 | 0.6 (0.3-2.3) | 1.1 (0.2-4.9) | | 0.14 |

[1]RR-risk ratio, comparing of characteristics (proportions) of viral and bacterial EM;

[2]P-value, comparing of characteristics of viral and bacterial EM;

[3]Mean (range);

[4]U-undefined;

[5]Median (interquartile range);

* There were no data for: 70 viral / 3 bacterial meningitis, 6 viral / 1 bacterial encephalitis, 5 viral / 3 bacterial meningoencephalitis (percentage was not calculated if white blood cells count was less than 100 in sight);

**Test was not done for 42 cases

**Table C. Use of antibacterial and antiviral treatment for acute encephalitis or meningitis patients.**

| Etiology | Number of cases | Antibacterial treatment | Antiviral treatment | Antibacterial treatment | p-value* |
|---|---|---|---|---|---|
| | N | n (%) | n (%) | Days (range) | |
| **Viral agents, Total** | **494** | 480 (97.2%) | 463 (93.7%) | 8.5 (1-23) | 0.4 |
| Enterovirus | 406 | 397 (97.8%) | 388 (95.6%) | 8.5 (1-23) | |
| Herpes simplex virus 1/2 | 71 | 67 (94.4%) | 64 (90.1%) | 8.9 (1-18) | |
| Varicella zoster virus | 13 | 12 (92.3%) | 11 (91.7%) | 8.2 (2-15) | |
| Rabies virus | 4 | 4 (100%) | 0 | 3 (2-4) | |
| **Bacterial Agents, Total** | **37** | 37 (100%) | 26 (70.3%) | 9.1 (2-20) | |
| *Neisseria meningitidis* | 21 | 21(100%) | 15 (71.4%) | 9.3 (2-20) | |
| *Streptococcus pneumoniae* | 10 | 10 (100%) | 8 (80.0%) | 9.2 (5-12) | |
| *Klebsiella pneumonia* | 1 | 1 (100%) | 0 | 4 | |
| *Klebsiella ozaenae* | 1 | 1 (100%) | 0 | 4 | |
| *Haemophilus influenzae* type b | 3 | 3 (100%) | 2 (66.7%) | 7.7 (6-9) | |
| *Listeria monocytogenes* | 1 | 1 (100%) | 1 (100.0%) | 17 | |
| Full negative | 19 | 19 (100%) | 16 (84.2%) | 8.6 (2-15) | |
| Not tested | 6 | 6 (100%) | 0 | 9.3 (2-13) | |
| **Total** | **556** | 542 (97.5%) | 511 (91.9%) | 8.6 (1-23)** | |

*Significance values comparing viral and bacterial meningitis by antibacterial treatment duration
** Data for antibacterial treatment duration were for 532 cases

**Table D. Susceptibility of *Neisseria meningitidis* (n=7) isolated from cerebrospinal fluid to antibacterial medicines.**

| Antibacterial medicines | No. Samples | No. Susceptible cultures | Percent |
|---|---|---|---|
| Ampicillin | 3 | 3 | 100 |
| Cefazolin | 7 | 6 | 86 |
| Ceftriaxone (CEF-3) | 5 | 4 | 80 |
| Cefepime | 6 | 4 | 67 |
| Ceftazidime | 3 | 2 | 67 |
| Penicillin | 7 | 4 | 57 |
| Ciprofloxacin | 6 | 3 | 50 |
| Gentamicin | 6 | 3 | 50 |
| Amikacin | 2 | 0 | 0 |
