# Supplementary material for: Etiology of acute meningitis and encephalitis from hospital-based surveillance in South Kazakhstan oblast, February 2017—January 2018
Source: PLoS One. 2021 May 14;16(5):e0251494. doi: 10.1371/journal.pone.0251494 (PMC8121361; doi:10.1371/journal.pone.0251494)
Supplement: S2 Appendix — (PDF) [file pone.0251494.s003.pdf]

Questionnaire № ☐☐☐ for epidemiological surveillance of encephalitis/meningitis case

The Questionnaire has been filled in:

(Name)______________________ Date of fill in ___/____________/ 20___

☐☐☐☐☐ Medical record №

☐☐☐☐☐☐☐ Case ID Code. (The first three full name letters, 2 first letters of the rayon/city, 2 last letters the birth year)

**Information about Respondent (if the patient is not the respondent)**	
Fill out this section if the patient is deceased or unresponsive. If the patient is the respondent, skip to the next section	
1. What was your relationship to the patient?<br>Parent<br>Spouse<br>Sibling<br>Child<br>Son-in-law or daughter-in-law<br>Parent-in-law<br>Friend or neighbor<br>Community leader<br>Health care worker<br>Other (specify)___________________	
2. **Hospitalization:** ☐ **Y** ☐ **N**	3. **Health facility:**
4. **Date of hospitalization: ____/________/ 20___y.**	5. **Diagnosis at admission (primary) :**

**Information about patient**	
6. **Date of birth: _____/_____/_______y.**	7. **Sex:** ☐**m ,** ☐**f**
8. **Residence address:**<br>**Oblast: ___________________, Rayon: ___________________, city/village: ______________________________**<br>***If it is impossible to give the exact address enter «Without permanent residence place»:** ☐	
9. **Permanently residing on the given territory:**<br>☐ **Y,** ☐ **N , if «N», then enter** ⟶	**Address, where arrived from**<br>____<br>____,<br>**When arrived: _____/_____/___________y**

10. **Ethnic group:** ________________________	11. **Education level:**<br>Illiterate<br>Below primary<br>Primary or middle<br>Secondary or high<br>College graduate<br>Postgraduate<br>Professional degree<br>Others (specify) ______________<br>Unknown
12. **Employment:**<br>Employed: ☐ **Y,** ☐ **N,** School student: ☐ **Y,** ☐ **N,** Higher school student: ☐ **Y,** ☐ **N,**<br>Attended kindergarten: ☐ **Y,** ☐ **N,** Pensioner:☐ **Y,** ☐ **N,** **Other** ________________________	
13. **Place of work/study** (if a pensioner or unemployed but works on his own farm or hired, indicate the workplace):	14. **Activity type (work as – for those working):**

Vaccination history

Data have been taken from the vaccination card: ☐ **Y,** ☐ **N** ☐ , data according to the oral information provided by a patient ☐ **Y,** ☐ **N**	
15. Vaccination against measles☐ **Y,** ☐ **N,** ☐ don't know, if Y, the number of vaccinations received ________,<br>Date of the last vaccination_____/_____/___________y<br>Vaccine ________________________	16. Vaccination against rubella: ☐ **Y,** ☐ **N,** ☐ don't know, if Y, then the number of vaccination received ________,<br>Date of the last vaccination<br>_____/_____/___________y<br>Vaccine________________________
17. Vaccination against Haemophilus influenza: ☐ **Y,** ☐ **N,** ☐ don't know, if Y, the number of vaccinations received ________,<br>Date of the last vaccination _____/_____/___________y<br>Vaccine ________________________	18. Vaccination against meningococcal infection ☐ **Y,** ☐ **N,** ☐ don't know, if Y, the number of vaccinations received ________,<br>Date of the last vaccination<br>_____/_____/___________y<br>Vaccine ________________________
19. Vaccination against pneumococcal infection: ☐ **Y,** ☐ **N,** ☐ don't know, if Y, the number of vaccinations received ________,<br>Date of the last vaccination _____/_____/___________y<br>Vaccine ________________________	20. Vaccination a month before the disease ☐ **Y,** ☐ **N,** ☐don't know, if Y, then against what infection ______________________________<br>Date of vaccination _____/_____/___________y<br>Vaccine ________________________
21. Vaccinations against tick-borne encephalitis: ☐ **Y,** ☐ **N,** ☐ don't know, if Y, the number of vaccinations received ________,<br>Date of the last vaccination _____/_____/___________y<br>Vaccine ________________________	
22. Comments important with regard to vaccination	

Clinical data (from the medical history, history card, talk with the attending doctor)

23. ***Date of the disease onset according to the history:*** ____/____________/ 20_______y.

24. Temperature at the time of the disease onset (according to the history) (______,___C), Date ____/________/ 20____y.	25. Maximum T^0C during the entire disease period (_____,__C), date____/________/ 20___y.

Symptoms and signs

26. Headache: ☐ Y, ☐ N Date of appearance ____/_____/ 20___y,
27. Vomiting: ☐ Y, ☐ N Date of appearance____/_____/ 20___y,
28. Diarrhea: ☐ Y, ☐ N Date of appearance____/_____/ 20___y,
29. Occipital muscle rigidity: ☐ Y, ☐ N Date of appearance____/_____/ 20___y,
30. Agitation: ☐ Y, ☐ N Date of appearance____/_____/ 20___y,
31. Confusional state: ☐ Y, ☐ N Date of appearance____/_____/ 20___y,
32. Retardation: ☐ Y, ☐ N Date of appearance____/_____/ 20___y,
33. Coma: ☐ Y, ☐ N Date of appearance____/_____/ 20___y,
34. Cramps: ☐ Y, ☐ N Date of appearance____/_____/ 20___y,
35. Bulging fontanel: ☐ Y, ☐ N Date of appearance____/_____/ 20___y,
36. Rash: ☐ Y, ☐ N Date of appearance____/_____/ 20___y, If "Yes", underline the type of rash: (spotted , popular, vesicular, pustular, nodular, hemorrhagic rash, other____________________________________(specify)
37. Muscle pain: ☐ Y, ☐ N Date of appearance____/_____/ 20___y,
38. Nausea: ☐ Y, ☐ N Date of appearance____/_____/ 20___y,
39. Lurch: ☐ Y, ☐ N Date of appearance____/_____/ 20___y,
40. Paresis / paralysis: ☐ Y, ☐ N Date of appearance____/_____/ 20___y,
41. Brudzinski's syndrome: ☐ Y, ☐ N Date of appearance____/_____/ 20___y,
42. Kernig's syndrome: ☐ Y, ☐ N Date of appearance____/_____/ 20___y,
43. Photophobia: ☐ Y, ☐ N Date of appearance____/_____/ 20___y,
44. Hydrophobia: ☐ Y, ☐ N Date of appearance____/_____/ 20___y,
45. Vesicular pharyngitis: ☐ Y, ☐ N Date of appearance____/_____/ 20___y,
46. Tonsillitis: ☐ Y, ☐ N Date of appearance____/_____/ 20___y,
47. Aggressive behavior toward others ☐ Y, ☐ N Date of appearance___/___/ 20___y,
48. Other symptoms: ☐ Y, ☐ N Date of appearance____/_____/ 20___y,
49. If Y, specify:____

**Hemorrhagic syndrome**	
50. **Petechial skin rash:** ☐Y , ☐ N, if «Y», then Date of appearance____/_________/ 20___y,<br><br>52. **Bleeding:** ☐Y, ☐ N, if «Y», then Date of appearance ____/_________/ 20___y,<br><br>53. **Positive bandage sign:** ☐Y, ☐ N, if «Y», then Date of appearance____/____/ 20___y	51. **Hemorrhage**: ☐Y, ☐ N, if «Y», then Date of appearance____/_______/ 20___y<br>Localization:<br>☐ Gastrointestinal,<br>☐ Metrorrhagia/ uterine bleeding<br>☐ Nasal bleeding<br>☐ Pulmonary hemorrhage<br>☐ Other (specify)______________________
54. **Platelet count on admission: ______________,** (10^9 /l)<br><br>**Date**______/____________/ 20____y	55. **Minimum number of platelets:**<br>**________________,** (10^9 /l)<br><br>**Date** _____/_____________/ 20____y
56. In the course of a month before the given disease were there other diseases (or suspicion of such):<br>☐ Y, ☐ N, if «Y», then specify	ARVI ☐ Y , ☐ N<br>Sore throat ☐ Y , ☐ N<br>Measles ☐ Y , ☐ N<br>Rubella ☐ Y , ☐ N<br>Varicella ☐ Y , ☐ N<br>Presence of primary suppurative focus ☐ Y , ☐ N, if «Yes» , then its localization<br>_______________________________________<br><br>Other disease _______________________<br><br>Date of the disease onset (or the period):<br>(c)____/__________/ 20___y<br>(по)____/__________/ 20___y

**LAB-CSF (CDL)**	
57. Date of CSF collection ____/_____/2017<br>58. Color____________________specify<br>59. Cytosis 10^6 /l ☐☐☐☐☐<br>60. neutrophils % ☐☐.☐☐<br>61. lymphocytes % ☐☐.☐☐<br>62. erythrocytes ☐☐☐☐<br>63. protein g/l ☐☐☐☐<br>64. sugar mmol/l ☐☐.☐☐<br>65. gram-negative bacilli (diplococci) ☐Y, ☐ N	66. Date of CSF collection ____/_____/2017 (5th day of the disease)<br>67. Cytosis 10^6 /l ☐☐☐☐☐<br>68. neutrophils % ☐☐.☐☐<br>69. lymphocytes % ☐☐.☐☐<br>70. erythrocytes ☐☐☐☐<br>71. Protein g/l ☐☐☐☐<br>72. sugar mmol/l ☐☐.☐☐<br>73. gram-negative bacilli (diplococci) ☐Y, ☐ N

LAB-BLOOD (CDL)

74. Date of blood collection _____/_______/2017<br><br>75. Leucocytes 10^9/l ☐☐☐☐<br>76. neutrophils % ☐☐.☐☐<br>77. lymphocytes % ☐☐.☐☐<br>78. ESR ☐☐☐☐<br>79. sugar mmol/l ☐☐.☐☐<br>80. Thick-blood diplococci ☐Y, ☐ N ☐ Not screened<br>81. Gram-negative bacilli(diplococci) ☐Y, ☐ N, ☐ Not screened	82. Date of blood collection _____/_______/2017 (5th day of disease)<br>83. Leucocytes 10^9 /l ☐☐☐☐<br>84. neutrophils % ☐☐.☐☐<br>85. lymphocytes % ☐☐.☐☐<br>86. sugar mmol/l ☐☐.☐☐<br>87. Thick-blood diplococci ☐Y, ☐ N ☐ Not screened<br>88. Gram-negative bacilli (diplococci) ☐Y, ☐ N, ☐ Not screened

Risk factors of case infection

89. **Have you had any contact** with the animals (during 2 weeks before the disease)<br>→<br>☐ Y, ☐ N, If «Y», then specify	**Type of contact:**<br>☐ pasturing,<br>☐ milking,<br>☐ riding on a horseback<br>☐ played with a dog<br>☐ shearing,<br>☐ participated in slaughtering animals<br>☐ participated in meat cutting<br>☐ other (specify) ______________________________<br><br>**Indicate the time of slaughtering:** _____/_____/2017y., if you do not know then indicate approximate dates (period) :
**Contact with a sick person:**<br>90. **Meningitis/encephalitis** (2 weeks before the disease) : ☐ Y, ☐ N,<br>91. **With hemorrhage** (2 weeks before the disease): ☐ Y, ☐ N, If «Y», then specify<br>92. **With measles** (3 weeks before the disease): ☐ Y, ☐ N<br>93. **With rubella** (3 weeks before the disease): ☐ Y, ☐ N<br>94. **Diseases with fever and rash** (3 weeks before the disease) : ☐ Y, ☐ N<br>95. **With ARVI:** ☐ Y, ☐ N<br>96. **With intestine infection (diarrhea):** ☐ Y, ☐ N<br>97. **A patient with other symptoms:** ☐ Y, ☐ N<br>**If Y, specify which**<br>___________________________________<br><br>→<br>If «Y», then specify	**Full name of a person you have had contact with:**<br>____<br>____<br><br>**Residence address:**<br>____<br>_<br>____<br>—<br>**Specify the date of contact or (period):**<br>(from)_____/_____/2017y., (to) _____/_____/2017y.,<br><br>**Specify what kind of contact (care) was that:**<br>____

98. **Possible contact in the hospital** (indicate any hospitalization in the course of 3 weeks **PRIOR** the given admission): → ☐ Y, ☐ N, If «Y», then specify	**Specify the hospital** _________________________________ and unit____________________________ With what diagnoses the patients were found in the ward/floor: • ARVI ☐Y, ☐N, ☐ unknown, • Meningitis ☐Y, ☐N, ☐ unknown , • encephalitis ☐Y, ☐N, ☐ unknown, • Other, specify ____ ______________________________________
99. **Have you been in the countriside** (2-3 weeks prior to the disease onset ☐Y, ☐N, ☐ unknown, If «Y», please, specify →	**Address, where have you been** ____ ____, **When have you been there:** **_____/_____/___________y** **swam in the open water body:** ☐Y, ☐N, ☐ unknown, if Y ,then specify the date _____/_____/201_y. And water body:_________________ drank water from the open water reservoir: ☐Y, ☐N, ☐ unknown, if Y , then specify the date _____/_____/201_y. And water reservoir:
100. **Have you swam in the basin** 3 weeks prior to the disease onset)**:** ☐Y, ☐N, ☐ unknown, If «Y», then specify →	Date _____/_____/201_y. Basin (specify): ____ Address:
101. **Other important information regarding risk factors for infection:**	

Information about bites

102. **Tick bite (in the course of 2weeks prior to fever development):** ☐Y, ☐N, if «N», then 103. **Have you contacted the** tick (with open skin parts): ☐ Y, ☐ N, → If «Y» for pp 97 or 98, then specify	**Date of the bite/contact:** **_____/________/ 201___y., _____/_________/ 201___y.** Specify the territory (address), where the tick bite has occurred (have contacted)

104. **Mosquito bite:** ☐Y, ☐N, **if «Y», then specify**	**Date of the bite:**<br><br>**_____/________/ 201___y., _____/_________/ 201___y.**
105. **Evidence of recent wounds:** ☐Y, ☐N, ☐DK	106. **Evidence of healed wounds:** ☐Y, ☐N, ☐DK
107. Have any family pets or livestock died during the 12 months prior to patient illness?<br>€ Yes (Date of death: ____ ____ / ____ ____ / ____ ____ ____ ____)<br>€ No<br>€ Unknown	108. Did **<deceased's name>** have any animal contact (bite, scratch, lick) with in the 12 months before the illness that led to death?<br>€ No<br>€ Yes<br>€ Unknown
If yes, please describe the animal contact events:	

	Animal 1	Animal 2	Animal 3
109. What date did <deceased> have contact with this animal?	__ __/ __ __/ __ __ __ __	__ __/ __ __/ __ __ __ __	__ __/ __ __/ __ __ __ __
110. What type of animal?	€ Dog<br>€ Cat<br>€ Bat<br>€ Livestock<br>€ Other: ____________	€ Dog<br>€ Cat<br>€ Bat<br>€ Livestock<br>€ Other: ____________	€ Dog<br>€ Cat<br>€ Bat<br>€ Livestock<br>€ Other: ____________
111. Was the animal owned?	€ Owned by deceased<br>€ Community owned<br>€ Unowned<br>€ Wild<br>€ Unknown	€ Owned by deceased<br>€ Community owned<br>€ Unowned<br>€ Wild<br>€ Unknown	€ Owned by deceased<br>€ Community owned<br>€ Unowned<br>€ Wild<br>€ Unknown
112. Did the animal have any signs of disease (Describe)?	□ YES □ NO □ Unknown	□ YES □ NO □ Unknown	□ YES □ NO □ Unknown
	€ Aggression<br>€ Paralysis<br>€ Biting<br>€ Hypersalivation<br>€ Lethargy<br>€ Other:	€ Aggression<br>€ Paralysis<br>€ Biting<br>€ Hypersalivation<br>€ Lethargy<br>€ Other:	€ Aggression<br>€ Paralysis<br>€ Biting<br>€ Hypersalivation<br>€ Lethargy<br>€ Other:
113. Is the animal alive today?<br>(If no, estimate date of death?)	□ YES □ NO □ Unknown<br>__ __/ __ __/ __ __ __ __	□ YES □ NO □ Unknown<br>__ __/ __ __/ __ __ __ __	□ YES □ NO □ Unknown<br>__ __/ __ __/ __ __ __ __

114. Was the animal observed for at least 10 days after the exposure?	€ YES, alive after 10 days<br>€ YES, died during observation<br>€ NO<br>€ Unknown	€ YES, alive after 10 days<br>€ YES, died during observation<br>€ NO<br>€ Unknown	€ YES, alive after 10 days<br>€ YES, died during observation<br>€ NO<br>€ Unknown
115. Was the animal tested for rabies?	€ YES, rabies positive<br>€ YES, rabies negative<br>€ NO<br>€ Unknown	€ YES, rabies positive<br>€ YES, rabies negative<br>€ NO<br>€ Unknown	€ YES, rabies positive<br>€ YES, rabies negative<br>€ NO<br>€ Unknown
116. Was the deceased bitten by this animal?	□ YES □ NO □ Unknown<br>*Location of bite:*<br>€ Head<br>€ Trunk<br>€ Upper limb<br>€ Hands<br>€ Lower limb<br>€ Genitalia<br>€ Other:	□ YES □ NO □ Unknown<br>*Location of bite:*<br>€ Head<br>€ Trunk<br>€ Upper limb<br>€ Hands<br>€ Lower limb<br>€ Genitalia<br>€ Other:	□ YES □ NO □ Unknown<br>*Location of bite:*<br>€ Head<br>€ Trunk<br>€ Upper limb<br>€ Hands<br>€ Lower limb<br>€ Genitalia<br>€ Other:
117. Did the deceased have other contact with the animal (i.e. licked, scratched)?	€ Scratch<br>€ Saliva contact with open wound or mucous membrane<br>€ Neural tissue contact with open wound or mucous membrane<br>€ Other:	€ Scratch<br>€ Saliva contact with open wound or mucous membrane<br>€ Neural tissue contact with open wound or mucous membrane<br>€ Other:	€ Scratch<br>€ Saliva contact with open wound or mucous membrane<br>€ Neural tissue contact with open wound or mucous membrane<br>€ Other:
118. What treatment did the patient receive for this contact?	€ Washed the wound<br>€ Sought medical care<br>€ Received rabies vaccination<br>€ Received immunoglobulin	€ Washed the wound<br>€ Sought medical care<br>€ Received rabies vaccination<br>€ Received immunoglobulin	€ Washed the wound<br>€ Sought medical care<br>€ Received rabies vaccination<br>€ Received immunoglobulin

119. **Other important information regarding infection risk factors:**

Treatment	
120. **Have been treated by antibiotics**<br><br>☐Y, ☐N, If «Y», then specify →	**Antibiotic 1__________________,**<br><br>Dose______________________________<br><br>Start date: _____/_________/20____y.<br><br>Date of the last dose taken: _____/_________/20____y.<br><br>**Antibiotic2__________________**<br><br>Dose______________________________<br><br>Prescription date: _____/_________/20____y.<br><br>Date of the last dose taken: _____/_________/20____y.<br><br>**Antibiotic 3__________________**<br><br>Dose______________________________<br><br>Date of prescription: _____/_________/20____y.<br><br>Date of the last dose taken: _____/_________/20____y.
121. Have been treated by gamma globulin<br>☐Y, ☐N, If «Y», then specify<br><br>→	**Gamma globulin** (Name) _________________________<br><br>Dose______________________________<br><br>Ate of prescription: _____/_________/20____y.<br><br>Date of the last dose taken: _____/_________/20____y.
122. Have been treated by antiviral drugs<br>☐Y, ☐N, If «Y», then specify<br><br>→	**Drug 1 (specify)** _________________________<br><br>Dose______________________________<br><br>Date of prescription: _____/_________/20____y.<br><br>Date of the last dose taken: _____/_________/20____y.<br><br>**Drug 2 (specify)** _________________________<br><br>Dose______________________________<br><br>Date of prescription: _____/_________/20____y.<br><br>Date of the last dose taken: _____/_________/20____y.

123. If the patient received antyrabies treatment, please specify the information:

Vaccine: ☐Y, ☐N, if Yes, name of vaccine____

- No. of injections ____________________
- Date started_____/_________/20____y
- Vaccination series completed?
  ☐Y, ☐N, ☐ DK

If Yes, date completed _____/_________/20____y

Rabies immunoglobulin: ☐Y, ☐N, if Yes

- No. of injections ____________________
- Date started_____/_________/20____y

124. Other important information on patient treatment

125. **Outcome of the disease:**

☐ **Has been discharged alive**, if «Y», then specify the date of the discharge:(dd/mm) _____/__________/20______y.,

Diagnose at discharge: ____

☐ **Died**, If «Y», then specify the date of the death (dd/mm) _____/________/20___y.,

Cause of death :____

☐ **The outcome of the disease is unknown**

Comments:

Laboratory tests:

PCR laboratory

126. **Spinal fluid** has been collected: ☐Y , ☐N, if «Y», then the date of collection __/_____/20___y.

**PCR tests**	**Spinal fluid testing:**	**Result**
PCR *Enterovirus:*	☐Y ☐N,	if Y then: ☐positive, ☐negative.
PCR *Neisseria meningitidis:*	☐Y ☐N,	if Y then: ☐ positive, ☐ negative
PCR *Haemophilus influenza type b:*	☐Y ☐N,	if Y then: ☐positive, ☐negative.
PCR *Streptococcus pneumoniae:*	☐Y ☐N,	if Y then: ☐positive, ☐negative.
PCR *Herpes simplex virus:*	☐Y ☐N,	if Y then: ☐positive, ☐negative.
PCR *Varicella zoster virus:*	☐Y ☐N,	if Y then: ☐positive, ☐negative.
PCR of *Listeria monocytogenes*:	☐Y ☐N,	if Y then:☐positive, ☐negative

Bacteriology laboratory

127. **Spinal fluid bacteriological test**: ☐Y ☐N, if then following pathogens have been isolated:

**Gram (-)bacteria:**	Test has been performed	Result
Neisseria meningitidis	☐Y , ☐N,	☐positive, ☐negative.
Escherichia coli	☐Y , ☐N,	☐ positive., ☐negative.
Haemophilus influenzae type b	☐Y , ☐N,	☐positive., ☐negative.
Klebsiella pneumoniae	☐Y , ☐N,	☐positive., ☐negative.
Pseudomonas spp	☐Y , ☐N,	☐positive., ☐negative.
Enterobacter spp	☐Y , ☐N,	☐positive., ☐negative.
Serratia spp	☐Y , ☐N,	☐positive., ☐negative.
Salmonella spp	☐Y , ☐N,	☐positive., ☐negative.
Other Gram (-), specify______________________	☐Y , ☐N,	☐positive., ☐negative.

**Gram (+) bacteria:**	Test has been performed	Result
Streptococcus pneumoniae	☐Y , ☐N,	☐positive., ☐negative.
Listeria monocytogenes	☐Y , ☐N,	☐positive., ☐negative.
Staphylococcus aureus	☐Y , ☐N,	☐positive., ☐negative.
Enterococcus spp	☐Y , ☐N,	☐positive., ☐negative.
GBS group B streptococcus	☐Y , ☐N,	☐positive., ☐negative.
GAS group A streptococcus	☐Y , ☐N,	☐positive., ☐negative.
Other Gram (+), specify ____________________________	☐Y , ☐N,	☐positive., ☐negative.

128. **Blood** has been collected: ☐Y , ☐N, if «Y», then date of collection ___/_____/20___y.

129. **Bacteriological test of blood**: ☐Y ☐N, if then following pathogens have been isolated:

**Gram (-)bacteria:**	Test has been performed	Result
Neisseria meningitidis	☐Y , ☐N,	☐positive, ☐negative.
Escherichia coli	☐Y , ☐N,	☐ positive., ☐negative.
Haemophilus influenzae	☐Y , ☐N,	☐positive., ☐negative.
Klebsiella pneumoniae	☐Y , ☐N,	☐positive., ☐negative.
Pseudomonas spp	☐Y , ☐N,	☐positive., ☐negative.
Enterobacter spp	☐Y , ☐N,	☐positive., ☐negative.
Serratia spp	☐Y , ☐N,	☐positive., ☐negative.
Salmonella spp	☐Y , ☐N,	☐positive., ☐negative.
Other Gram (-), specify __________________________	☐Y , ☐N,	☐positive., ☐negative.
**Gram (+) bacteria:**	Test has been performed	Result
Streptococcus pneumoniae	☐Y , ☐N,	☐positive., ☐negative.
Listeria monocytogenes	☐Y , ☐N,	☐positive., ☐negative.
Staphylococcus aureus	☐Y , ☐N,	☐positive., ☐negative.
Enterococcus spp	☐Y , ☐N,	☐positive., ☐negative.
GBS group B streptococcus	☐Y , ☐N,	☐positive., ☐negative.
GAS group A streptococcus	☐Y , ☐N,	☐positive., ☐negative.
Other Gram (+)	☐Y , ☐N,	☐positive., ☐negative.
