# Supplementary material for: Etiology of acute meningitis and encephalitis from hospital-based surveillance in South Kazakhstan oblast, February 2017—January 2018
Source: PLoS One. 2021 May 14;16(5):e0251494. doi: 10.1371/journal.pone.0251494 (PMC8121361; doi:10.1371/journal.pone.0251494)
Supplement: S3 Appendix — (PDF) [file pone.0251494.s004.pdf]

Анкета № □□□ для эпидемиологического расследования случая энцефалита/менингита

Анкету заполнил(ФИО)_____________________ Дата заполнения ___/____________/ 20___г

□□□□□ № истории болезни

□□□□□□□ Код. Случая (первые три буквы ФИО, 2 первые буквы района/города, 2 последние цифры года рождения)

**Информация о респонденте (если пациент не является респондентом)**
Заполните эту секцию, если пациент умер или не может отвечать. Если пациент отвечает сам, то перепрыгните на вопрос 3.
1. Отношение к пациенту?<br>Родители<br>Супруги<br>Брать/сестра<br>Ребенок родной<br>Усыновленные дети<br>Родители мужа/жены<br>Друзья или соседи<br>Глава поселка<br>Медработник<br>Другое (укажите)__________________

2. **Госпитализация:** □ **да** □ **нет**	3. **Лечебная организация :**
4. **Дата госпитализации: ____/________/ 20___г.**	5. **Диагноз при поступлении :**
***Паспортные данные**	
6. **Дата рождения:**<br>_____/_____/__________г	7. **Пол:** □**м ,** □**ж**
8. **Адрес проживания:**<br>**Область:** __________________, **Район:** __________________, **город/село:** ____________________________<br>***Если невозможно указать определенный адрес, то укажите «Без определенного места жительства»:** □	

9. **Постоянно проживает на данной территории:** ☐ **да,** ☐ **нет, если «НЕТ», то укажите** →	**адрес, откуда приехал** ____ ____, **когда приехал:** _____/_____/___________**г**

10. Занятость:
Работа имеется: ☐ да, ☐ нет, Учится в школе: ☐ да, ☐ нет,
Учится в ВУЗе: ☐ да, ☐ нет, Пенсионер: ☐ да, ☐ нет,
Посещает ДДУ: ☐ да, ☐ нет, Другое ________________________

11. **Национальность** ________________	12. **Уровень образования:** Неграмотный; Ниже начального; Начальное или ниже среднего; Среднее /среднеспециальное; Высшее образование; Магистратура; Техникум/ПТУ; Другое (уточнить) ______________; неизвестно
13. **Место работы/учебы** (если пенсионер или безработный, но работает в своем хозяйстве или по найму, указать как место работы):	14. **Род деятельности** (кем работает - для работающих):

*Прививочный анамнез

Данные взяты из прививочной карты: ☐ да, ☐ нет, данные взяты со слов больного ☐ да, ☐ нет

15. Привки против кори: ☐ да, ☐ нет, ☐ не знаю, если да, то число полученных прививок ________, Дата последней прививки_____/_____/___________г Вакцина ________________________	16. Привки против краснухи: ☐ да, ☐ нет, ☐ не знаю, если да, то число полученных прививок ________, Дата последней прививки_____/_____/___________г Вакцина_________________________
17. Привки против гемофильной палочки инфлюэнцы: ☐ да, ☐ нет, ☐ не знаю если да, то число полученных прививок ________, Дата последней прививки_____/_____/___________г Вакцина ________________________	18. Привки против менингококковойинфекции: ☐ да, ☐ нет, ☐ не знаю если да, то число полученных прививок ________, Дата последней прививки_____/_____/___________г Вакцина ________________________
19. Привки против пневмококковойинфекции: ☐ да, ☐ нет, ☐ не знаю, если да, то число полученных прививок ________, Дата последней прививки_____/_____/___________г Вакцина ________________________	20. Получение вакцины за месяц до заболевания☐ да, ☐ нет, ☐ не знаю,если да, то против какой инфекции______________________________ Дата прививки_____/_____/___________г Вакцина ________________________
21. Привки против клещевого энцефалита: ☐ да, ☐ нет, ☐ не знаю, если да, то число полученных прививок ________, Дата последней прививки_____/_____/___________г	22. Комментарии важные в отношении вакцинации

Вакцина ________________________	
***Клинические данные** (из анамнеза, истории болезни, беседы с лечащим врачом)	
23. ***Дата начала заболевания из анамнеза:*** ____/____________/ 20_______г.	
24. Температура на момент начала заболевания (из анамнеза) (______,___С), дата ____/________/ 20____г.	25. Максимальная T^0C за весь период болезни (_____,__С), дата____/________/ 20___г.

***Симптомы и признаки**

26. Головная боль: ☐ да, ☐ нет Дата появления____/_____/ 20___г ,
27. Рвота: ☐ да, ☐ нет Дата появления____/_____/ 20___г ,
28. Диарея: ☐ да, ☐ нет Дата появления____/_____/ 20___г ,
29. Регидность з/м ☐ да, ☐ нет Дата появления____/_____/ 20___г ,
30. Возбуждение ☐ да, ☐ нет Дата появления____/_____/ 20___г ,
31. Спутанное сознание ☐ да, ☐ нет Дата появления____/_____/ 20___г ,
32. Заторможен-ность ☐ да, ☐ нет Дата появления____/_____/ 20___г ,
33. Кома ☐ да, ☐ нет Дата появления____/_____/ 20___г ,
34. Судороги ☐ да, ☐ нет Дата появления____/_____/ 20___г ,
35. Выпяч. Родничка ☐ да, ☐ нет Дата появления____/_____/ 20___г ,
36. Сыпь ☐ да, ☐ нет Дата появления____/_____/ 20___г , если да, то какая, подчеркнуть : (пятнистая, папулезная, везикулезная, пустуллезная, узелковая, эритематозная, геморрагическая), другая_____________________________________(указать)

37. Мышечная боль: ☐ да, ☐ нет Дата появления____/_____/ 20___г ,
38. Тошнота: ☐ да, ☐ нет Дата появления____/_____/ 20___г ,
39. Шаткая походка ☐ да, ☐ нет Дата появления____/_____/ 20___г ,
40. Парез/паралич ☐ да, ☐ нет Дата появления____/_____/ 20___г ,
41. С-м Брудзинского ☐ да, ☐ нет Дата появления____/_____/ 20___г ,
42. С-м Кернига ☐ да, ☐ нет Дата появления____/_____/ 20___г ,
43. Светобоязнь ☐ да, ☐ нет Дата появления____/_____/ 20___г ,
44. Водобоязнь ☐ да, ☐ нет Дата появления____/_____/ 20___г ,
45. Везикулярный фарингит ☐ да, ☐ нет Дата появления____/_____/ 20___г ,
46. Тонзилит ☐ да, ☐ нет Дата появления____/_____/ 20___г ,
47. Агрессивное поведение ☐ да, ☐ нет Дата появления____/_____/ 20___г ,

48. Другие симптомы: ☐ да, ☐ нет Дата появления____/_____/ 20___г ,
49. Если Да, указать какие:____

*Геморрагический синдром

50. **Петехиальная сыпь:** ☐да , ☐ нет, если «Да», то Дата появления____/_________/ 20___г,

51. **Кровотечение**: ☐да, ☐ нет, если «Да», то Дата ____/__________/ 20___г
Локализация:
☐ Желудочно-кишечное,
☐ Маточное
☐ Носовое
☐ Легочное
☐ Другое (указать)______________________

52. **Кровоизлияния:** ☐да, ☐ нет, если «Да», то Дата появления ____/_________/ 20___г,

53. **Положительный симптом жгута:** ☐да, ☐ нет, если «Да», то Дата появления____/_______/ 20___г

54. **Число тромбоцитов при поступлении:** ______________**, (кл/мм³)**
Дата______/____________/ 20____г

55. **Минимальное число тромбоцитов:** ________________**, (кл/мм³)**
Дата_____/_____________/ 20____г

56. В течение месяца до настоящего заболевания были ли другие заболевания (или подозрение на них):
☐ да, ☐ нет, Если «ДА», то укажите →

ОРВИ ☐ да , ☐ нет
Ангина ☐ да , ☐ нет
Корь ☐ да , ☐ нет
Краснуха ☐ да , ☐ нет
Ветряная оспа ☐ да , ☐ нет
Наличие первичного гнойного очага ☐ да , ☐ нет, если да, то его локализация
____

Другое заболевание______________________

Дата начала заболевания (или период): (с)____/_________/ 20___г
(по)____/__________/ 20___г

Лаборатория СПЖ (КДЛ) *LAB-CSF

57. Дата забора____/_____/2017 при поступлении
58. Цвет____________________указать
59. Цитоз (10^6/л) ☐☐☐☐☐
60. нейтрофилы % ☐☐.☐☐
61. лимфоциты % ☐☐.☐☐
62. эритроциты ☐☐☐☐
63. белок г/л ☐☐☐☐
64. сахар ммоль/л ☐☐.☐☐
65. Гр отр.палочки(диплококки) ☐да, ☐ нет

66. Дата ____/_____/2017 (5-й день заболевания)
67. цитоз (10^6/л) ☐☐☐☐☐
68. нейтрофилы % ☐☐.☐☐
69. лимфоциты % ☐☐.☐☐
70. эритроциты ☐☐☐☐
71. белок г/л ☐☐☐☐
72. сахар ммоль/л ☐☐.☐☐
73. Гр отр.палочки(диплококки) ☐да, ☐ нет

Лаборатория крови (КДЛ) *LAB-BLOOD	
74. Дата забора_____/_______/20 г. при поступлении<br><br>75. лейкоциты 10^9/л ☐☐☐☐<br>76. нейтрофилы % ☐☐.☐☐<br>77. лимфоциты % ☐☐.☐☐<br>78. СОЭ ☐☐☐☐<br>79. сахар ммоль/л ☐☐.☐☐<br>80. толстая капля диплококки ☐да, ☐ нет ☐ не обследован<br>81. Гр отр.палочки(диплококки) ☐да, ☐ нет ☐ не обследован	82. Дата _____/_______/20 г. (5-й день заболевания)<br>83. лейкоциты 10^9/л ☐☐☐☐<br>84. нейтрофилы % ☐☐.☐☐<br>85. лимфоциты % ☐☐.☐☐<br>86. сахар ммоль/л ☐☐.☐☐<br>87. толстая капля диплококки ☐да, ☐ нет ☐ не обследован<br>88. Гр отр.палочки (диплококки) ☐да, ☐ нет ☐ не обследован
***Факторы риска заражения случая**	
89. **Имелся ли любой контакт с** животными (за 2 недели до заболевания)<br><br>☐ да, ☐ нет, Если «ДА», то укажите<br><br>→	**тип контакта:**<br>☐ выпас,<br>☐ дойка,<br>☐ ездил верхом на лошади,<br>☐ играл с собакой,<br>☐ стрижка,<br>☐ участвовали в забое животных<br>☐ участвовал(а) в разделке мяса животных<br>☐ другое (укажите) ______________________________<br><br>**Укажите дату забоя:** _____/_____/2017г., если не знаете, то укажите даты (период) ориентировочно:
**Контакт с больным человеком:**<br>90. **Менингитом/энцефалитом** (за 2 недели до заболевания) : ☐ да, ☐ нет,<br>91. **С кровотечением** (за 2 недели до заболевания):<br>☐ да, ☐ нет, Если «ДА», то укажите<br>92. **С корью** (за 3 недели до заболевания):<br>☐ да, ☐ нет<br>93. **С краснухой** (за 3 недели до заболевания):<br>☐ да, ☐ нет<br>94. **Заболеванием с температурой и сыпью** (за 3 недели до заболевания) : ☐ да, ☐ нет<br>95. **С ОРВИ:** ☐ да, ☐ нет<br>96. **С кишечной инфекцией (диарея):** ☐ да, ☐ нет<br>97. **Больной с другими симптомами:** ☐ да, ☐ нет<br>**Если да, укажите с какими**___________________________________<br><br>→<br>Если «ДА», то укажите	**С кем был контакт ФИО:**<br><br>____<br><br>____<br><br>**Адрес проживания:**<br><br>____<br><br>____<br><br>**Укажите дату контакта или (период):**<br><br>(с)_____/_____/2017г., (по) _____/_____/2017г.,<br><br>**Укажите, в чем заключался контакт (уход):**<br>____

98. **Возможный контакт в больнице** (укажите любую госпитализацию в течение 3 недель **ДО** данного поступления):<br>☐ да, ☐ нет, Если «ДА», то укажите →	**Укажите больницу** ________________ и<br><br>отделение______________<br><br>С какими диагнозами лежали больные в палате/этаже:<br>• ОРВИ ☐да, ☐нет, ☐ неизвестно,<br>• Менингит ☐да, ☐нет, ☐ неизвестно,<br>• Энцефалит ☐да, ☐нет, ☐ неизвестно,<br>• Другие, укажите<br>______________________<br>______________
99. **Выезжал на природу** (за 2-3 недели до заболевания)**:** ☐да, ☐нет, ☐ неизвестно,<br><br>Если «ДА», то укажите →	**адрес, куда выезжал**______________________<br><br>______________________,<br><br>**когда выезжал: _____/_____/___________г**<br><br>**купался в открытом водоеме:** ☐да, ☐нет, ☐ неизвестно, если Да , то укажите дату _____/_____/201_г.<br>и водоем:_________<br><br>**пил ли воду из открытого водоема**: ☐да, ☐нет, ☐ неизвестно если Да , то укажите дату _____/_____/201_г. и водоем:
100. **Купался ли в бассейне** (за 3 недели до заболевания)**:** ☐да, ☐нет, ☐ неизвестно,<br><br>Если «ДА», то укажите →	Дату _____/_____/201_г.<br><br>Бассейн (название):<br>______________________<br><br>Адрес:
101. **Другая важная информация в отношении факторов риска заражения:**	
***Информация об укусах клеща**	
102. **Укус клеща (в течение 2-х недель до появления лихорадки):** ☐да, ☐нет, если «НЕТ», то<br><br>103. **Был ли контакт с** клещом (открытых участков кожи): ☐ да, ☐ нет,<br>если «ДА» на пп.97 или 98, то укажите →	**Дата(ы) укуса/контакта:**<br><br>**_____/________/ 201___г., _____/_________/ 201___г.**<br><br>Укажите территорию (адрес), где произошел укус клеща (был контакт)

104. **Укус комара:** ☐да, ☐нет, **если «ДА», то укажите**	**Дата укуса:**<br>**_____/________/ 201___г., _____/_________/ 201___г.**

***Укус/контакт с животными**

105. **Имеются ли свежие раны:**<br>☐Да, ☐Нет, ☐Неизвестно	106. **Имеются ли зажившие раны:**<br>☐Да, ☐Нет, ☐Неизвестно
107. Имели место случаи смерти среди собак (других питомцев) или домашних животных в течение 12 мес до заболевания пациента?<br>€ Да (Дата смерти: ____ ____ / ____ ____ / ____ ____ ____ ____)<br>€ Нет<br>€ Неизвестно	108. Имел ли пациент контакт с любым животным за 12 месяцев до заболевания?<br>укус, ☐Да, ☐Нет, ☐Неизвестно<br>оцарапывание, ☐Да, ☐Нет, ☐Неизвестно<br>ослюнение ☐Да, ☐Нет, ☐Неизвестно

Если, да, то опишите подробно контакт:

	**Животное 1**	**Животное 2**	**Животное 3**
109. Дата контакта с животным?	__ __ / __ __ / __ __ __ __	__ __ / __ __ / __ __ __ __	__ __ / __ __ / __ __ __ __
110. Вид животного?	€ собака<br>€ кошка<br>€ летучая мышь<br>€ домашний скот<br>€ другие: __________	€ собака<br>€ кошка<br>€ летучая мышь<br>€ домашний скот<br>€ другие: __________	€ собака<br>€ кошка<br>€ летучая мышь<br>€ домашний скот<br>€ другие: __________
111. Владелец животного?	€ заболевший<br>€ соседи<br>€ без хозяина<br>€ дикое животное<br>€ неизвестно	€ заболевший<br>€ соседи<br>€ без хозяина<br>€ дикое животное<br>€ неизвестно	€ заболевший<br>€ соседи<br>€ без хозяина<br>€ дикое животное<br>€ неизвестно
112. Были ли у животного признаки заболевания (опишите)?	☐Да, ☐Нет, ☐ Неизвестно	☐Да, ☐Нет, ☐ Неизвестно	☐Да, ☐Нет, ☐ Неизвестно
	€ агрессия<br>€ паралич<br>€ кусалось<br>€ слюнотечение<br>€ заторможенность<br>€ другое:	€ агрессия<br>€ паралич<br>€ кусалось<br>€ слюнотечение<br>€ заторможенность<br>€ другое:	€ агрессия<br>€ паралич<br>€ кусалось<br>€ слюнотечение<br>€ заторможенность<br>€ другое:
113. На сегодня животное живое?<br>(Если нет, примерная дата	☐Да, ☐Нет, ☐ Неизвестно __ __ / __ __ / __ __ __ __	☐Да, ☐Нет, ☐ Неизвестно __ __ / __ __ / __ __ __ __	☐Да, ☐Нет, ☐ Неизвестно __ __ / __ __ / __ __ __ __

смерти?)			
114. Наблюдали ли за животным в течение 10 дней после укуса?	€ Да, живо после 10 дней наблюдения<br>€ Да, умерло в течение наблюдения<br>€ Нет<br>€ Неизвестно	€ Да, живо после 10 дней наблюдения<br>€ Да, умерло в течение наблюдения<br>€ Нет<br>€ Неизвестно	€ Да, живо после 10 дней наблюдения<br>€ Да, умерло в течение наблюдения<br>€ Нет<br>€ Неизвестно
115. Было животное тестировано на бешенство?	€ Да, положительно<br>€ Да, отрицательно<br>€ Нет<br>€ Неизвестно	€ Да, положительно<br>€ Да, отрицательно<br>€ Нет<br>€ Неизвестно	€ Да, положительно<br>€ Да, отрицательно<br>€ Нет<br>€ Неизвестно
116. Если был укус животного?	☐Да, ☐Нет, ☐ Неизвестно<br>*Укажите локализацию:*<br>€ Голова<br>€ Туловище<br>€ Верхние конечности<br>€ Кисти<br>€ Нижние конечности<br>€ Гениталии<br>€ другое:	☐Да, ☐Нет, ☐ Неизвестно<br>*Укажите локализацию:*<br>€ Голова<br>€ Туловище<br>€ Верхние конечности<br>€ Кисти<br>€ Нижние конечности<br>€ Гениталии<br>€ другое:	☐Да, ☐Нет, ☐ Неизвестно<br>*Укажите локализацию:*<br>€ Голова<br>€ Туловище<br>€ Верхние конечности<br>€ Кисти<br>€ Нижние конечности<br>€ Гениталии<br>€ другое:
117. Был ли другой тип контакта (ослюнение, оцарапывание)?	€ Оцарапывание<br>€ Ослюнение открытой раны/слизистых<br>€ Контакт нервной ткани с открытой раной/слизистыми<br>€ другое:	€ Оцарапывание<br>€ Ослюнение открытой раны/слизистых<br>€ Контакт нервной ткани с открытой раной/слизистыми<br>€ другое:	€ Оцарапывание<br>€ Ослюнение открытой раны/слизистых<br>€ Контакт нервной ткани с открытой раной/слизистыми<br>€ другое:
118. Какое лечение было получено по поводу этого контакта/укуса?	€ Промывание раны<br>€ Антирабический иммуноглобуллин<br>Дата_____/________/ 17г.<br>€ Антирабическая вакцина<br>Дата_____/________/ 17г.<br>Курс______прививок	€ Промывание раны<br>€ Антирабический иммуноглобуллин<br>Дата_____/________/ 17г.<br>€ Антирабическая вакцина<br>Дата_____/________/ 17г.<br>Курс______прививок	€ Промывание раны<br>€ Антирабический иммуноглобуллин<br>Дата_____/________/ 17г.<br>€ Антирабическая вакцина<br>Дата_____/________/ 17г.<br>Курс______прививок

119. **Другая важная информация относительно факторов риска заболевания:**

*Лечение	
120. **Лечение анибиотиками проводилось**<br>☐да, ☐нет, Если «Да», то укажите →	**Антибиотик 1__________________,**<br>Доза______________________________<br>Дата назначения: _____/_________/20____г.<br>Дата последнего приема: _____/_________/20____г.<br>**Антибиотик 2__________________**<br>Доза______________________________<br>Дата назначения: _____/_________/20____г.<br>Дата последнего приема: _____/_________/20____г.<br>**Антибиотик 3__________________**<br>Доза______________________________<br>Дата назначения: _____/_________/20____г.<br>Дата последнего приема: _____/_________/20____г.
121. Лечение гаммаглобулином проводилось<br>☐да, ☐нет, Если «Да», то укажите →	**Название** _________________________<br>Доза______________________________<br>Дата назначения: _____/_________/20____г.<br>Дата последнего приема: _____/_________/20____г.
122. Лечение противовирусными препаратами проводилось<br>☐да, ☐нет, Если «Да», то укажите →	**Название 1** _________________________<br>Доза______________________________<br>Дата назначения: _____/_________/20____г.<br>Дата последнего приема: _____/_________/20____г.<br>**Название 2** _________________________<br>Доза______________________________<br>Дата назначения: _____/_________/20____г.<br>Дата последнего приема: _____/_________/20____г.

123. Если больной получал вакцинацию против бешенства, пожалуйста уточните:

Вакцинация: ☐ да, ☐ нет, Если «Да», то укажите, название вакцины

____

- Число инъекций ____________________
- Дата начала_____/_________/20____г.
- Схема вакцинации закончена?

☐ да, ☐ нет, ☐ неизвестно

Если «Да», то укажите дату завершения _____/_________/20____г.

Антирабический иммуноглобулин применялся: ☐ да, ☐ нет, Если «Да», то укажите

- Число инъекций ____________________
- Дата начала_____/_________/20____г.

124. Другая важная информация по лечению пациента

125. **Исход заболевания:**

☐ **Выписан живым**, Если «ДА», то дата выписки:(дд/мм) _____/__________/20______г.,

Диагноз при выписке: ____

☐ **Умер**, Если «ДА», то дата смерти:(дд/мм) _____/________/20___г.,

Причина смерти:____

☐ **Исход заболевания не известен**

Комментарии:

Лабораторное исследования:

126. **Забор ликвора** произведен: ☐да , ☐нет, если «Да», то дата забора __/_____/20___г.

**ПЦР тест**	**Проведение теста из ликвора:**	**Результат**
ПЦР *Enterovirus:*	☐да ☐нет,	если да то: ☐положительный, ☐отриц.
ПЦР *Neisseria meningitidis:*	☐да ☐нет,	если да то: ☐положительный, ☐отриц.
ПЦР *Haemophilus influenza:*	☐да ☐нет,	если да то: ☐положительный, ☐отриц.
ПЦР *Streptococcus pneumoniae*:	☐да ☐нет,	если да то: ☐положительный, ☐отриц.
ПЦР *Herpes simplex virus*:	☐да ☐нет,	если да то: ☐положительный, ☐отриц.
ПЦР *Varicella zoster virus*:	☐да ☐нет,	если да то: ☐положительный, ☐отриц.
ПЦР *Listeria monocytogenes*:	☐да ☐нет,	если да то: ☐положительный, ☐отриц.

127. **Бактериологическое исследование ликвора**: ☐да ☐нет, если да то, выдлены ли следуюшие возбудители:

**Грам (-) бактерии:**	Анализ произведен	Результат
Neisseria meningitidis	☐да , ☐нет,	☐положит., ☐отриц.
Escherichia coli	☐да , ☐нет,	☐положит., ☐отриц.
Haemophilus influenzae	☐да , ☐нет,	☐положит., ☐отриц.
Klebsiella pneumoniae	☐да , ☐нет,	☐положит., ☐отриц.
Pseudomonas spp	☐да , ☐нет,	☐положит., ☐отриц.
Enterobacter spp	☐да , ☐нет,	☐положит., ☐отриц.
Serratia spp	☐да , ☐нет,	☐положит., ☐отриц.
Salmonella spp	☐да , ☐нет,	☐положит., ☐отриц.
Другие Грам (-), укажите___________________	☐да , ☐нет,	☐положит., ☐отриц.
**Грам (+) бактерии:**	Анализ произведен	Результат
Streptococcus pneumoniae	☐да , ☐нет,	☐положит., ☐отриц.
Listeria monocytogenes	☐да , ☐нет,	☐положит., ☐отриц.
Staphylococcus aureus	☐да , ☐нет,	☐положит., ☐отриц.

Enterococcus spp	☐да , ☐нет,	☐положит., ☐отриц.
GBS group B streptococcus	☐да , ☐нет,	☐положит., ☐отриц.
GAS group A streptococcus	☐да , ☐нет,	☐положит., ☐отриц.
Другие Грам (+), укажите ________________________	☐да , ☐нет,	☐положит., ☐отриц.

128. **Забор крови** произведен: ☐да , ☐нет, если «Да», то Дата забора ___/_____/20___г.

129. **Бактериологическое исследование крови**: ☐да ☐нет, если да то, выдлены ли следуюшие возбудители:

**Грам (-) бактерии:**	Анализ произведен	Результат
Neisseria meningitidis	☐да , ☐нет,	☐положит., ☐отриц.
Escherichia coli	☐да , ☐нет,	☐положит., ☐отриц.
Haemophilus influenzae	☐да , ☐нет,	☐положит., ☐отриц.
Klebsiella pneumoniae	☐да , ☐нет,	☐положит., ☐отриц.
Pseudomonas spp	☐да , ☐нет,	☐положит., ☐отриц.
Enterobacter spp	☐да , ☐нет,	☐положит., ☐отриц.
Serratia spp	☐да , ☐нет,	☐положит., ☐отриц.
Salmonella spp	☐да , ☐нет,	☐положит., ☐отриц.
Другие Грам (-), укажите ________________________	☐да , ☐нет,	☐положит., ☐отриц.
**Грам (+) бактерии:**	Анализ произведен	Результат
Streptococcus pneumoniae	☐да , ☐нет,	☐положит., ☐отриц.
Listeria monocytogenes	☐да , ☐нет,	☐положит., ☐отриц.
Staphylococcus aureus	☐да , ☐нет,	☐положит., ☐отриц.
Enterococcus spp	☐да , ☐нет,	☐положит., ☐отриц.
GBS group B streptococcus	☐да , ☐нет,	☐положит., ☐отриц.
GAS group A streptococcus	☐да , ☐нет,	☐положит., ☐отриц.
Другие Грам (+), укажите______________	☐да ☐нет,	☐положит., ☐отриц.
