# Supplementary material for: Etiology of acute meningitis and encephalitis from hospital-based surveillance in South Kazakhstan oblast, February 2017—January 2018
Source: PLoS One. 2021 May 14;16(5):e0251494. doi: 10.1371/journal.pone.0251494 (PMC8121361; doi:10.1371/journal.pone.0251494)
Supplement: S1 Text — (PDF) [file pone.0251494.s005.pdf]

Этиология острых менингитов и энцефалитов по результатам госпитального эпиднадзора в Южно-Казахстанской области Казахстана, февраль 2017 г. - январь 2018 г.

Екатерина Бумбуриди, Гульмира Утебергенова, Бахтыгали Ережепов, Нурсулу Бердиярова, Калдикуль Кульжанова, Дженнифер Хэд, Дафна Моффетт, Даниэль Сингер, Паван Ангра, Тони Вистлер, Джеймс Сейджвар

Энцефалит и менингит (ЭМ) являются тяжелыми инфекционными заболеваниями центральной нервной системы и часто характеризуются высокой заболеваемостью и смертностью. Этиология ЭМ в Казахстане четко не определена, поэтому с 1 февраля 2017 года по 31 января 2018 года мы проводили госпитальный синдромальный эпиднадзор за ЭМ в Шымкентской городской больнице, куда госпитализировались также больные, направленные из районных больницах Южно-Казахстанской области. Госпитализированные случаи ЭМ, соответствующие стандартному определению случая и давшие согласие на участие, были взяты в исследование. Кровь и спинномозговая жидкость (СМЖ) были исследованы на культуру бактерий и СМЖ была протестирована в ПЦР на четыре бактерии и три вируса с использованием каскадного алгоритма. Из 556 зарегистрированных пациентов: 494 имели вирусную этиологию, (включая 4 вероятных случая бешенства), 37 бактериальную, в 19 случаях не было выявлено ни одного патогена и 6 случаев не были протестированы. Наиболее распространенными патогенами были энтеровирусы (73,0%, n= 406 случаев), за которым следовали случаи вируса простого герпеса (12,8%, n=71) и случаи менингококка (3,8%, n=21). Показатели заболеваемости энтеровирусными и менингококковыми ЭМ составили соответственно 14,5 и 0,7 случая на 100 000 населения. Показатели заболеваемости бактериальными менингитами при использовании обоих методов - ПЦР и культуры были в 3-5 раз выше, чем без ПЦР. Антибактериальные препараты были использованы для лечения 97,2% (480/494) пациентов, которые имели вирусную этиологию ЭМ. Включение ПЦР в рутинную лабораторную диагностику ЭМ улучшит идентификацию патогенов, позволит исключить недооценку показателей заболеваемости и поможет избежать ненужного лечения антибиотиками.
